# Iron Chelation in Soil: Scalable Biotechnology for Accelerating Carbon Dioxide Removal by Enhanced Rock Weathering

**DOI:** 10.1021/acs.est.3c10146

**Published:** 2024-06-24

**Authors:** Dimitar Z. Epihov, Steven A. Banwart, Steve P. McGrath, David P. Martin, Isabella L. Steeley, Vicky Cobbold, Ilsa B. Kantola, Michael D. Masters, Evan H. DeLucia, David J. Beerling

**Affiliations:** † Levehulme Centre for Climate Change Mitigation, School of Biosciences, University of Sheffield, Sheffield S10 2TN, U.K.; ‡ Global Food and Environment Institute, University of Leeds, Leeds LS2 9JT, U.K.; § School of Earth and Environment, University of Leeds, Leeds LS2 9JT, U.K.; ∥ Sustainable Soils and Crops, Rothamsted Research, Harpenden AL5 2JQ, U.K.; ⊥ Institute for Sustainability, Energy, and Environment, University of Illinois at Urbana−Champaign, Urbana, Illinois 61801, United States

**Keywords:** chelator, chelating agent, enhanced weathering, carbon dioxide removal, carbon capture, siderophore, EDDHA, basalt, biotechnology

## Abstract

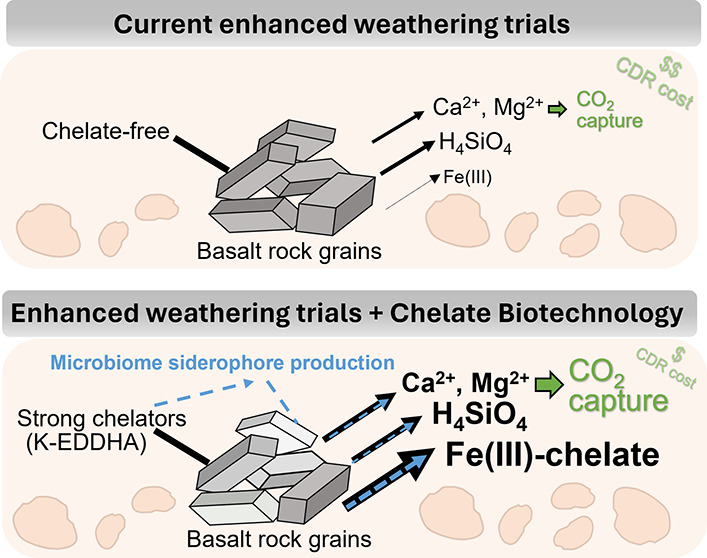

Enhanced rock weathering (EW) is an emerging atmospheric carbon dioxide removal (CDR) strategy being scaled up by the commercial sector. Here, we combine multiomics analyses of belowground microbiomes, laboratory-based dissolution studies, and incubation investigations of soils from field EW trials to build the case for manipulating iron chelators in soil to increase EW efficiency and lower costs. Microbial siderophores are high-affinity, highly selective iron (Fe) chelators that enhance the uptake of Fe from soil minerals into cells. Applying RNA-seq metatranscriptomics and shotgun metagenomics to soils and basalt grains from EW field trials revealed that microbial communities on basalt grains significantly upregulate siderophore biosynthesis gene expression relative to microbiomes of the surrounding soil. Separate *in vitro* laboratory incubation studies showed that micromolar solutions of siderophores and high-affinity synthetic chelator (ethylenediamine-*N*,*N*′-bis-2-hydroxyphenylacetic acid, EDDHA) accelerate EW to increase CDR rates. Building on these findings, we develop a potential biotechnology pathway for accelerating EW using the synthetic Fe-chelator EDDHA that is commonly used in agronomy to alleviate the Fe deficiency in high pH soils. Incubation of EW field trial soils with potassium-EDDHA solutions increased potential CDR rates by up to 2.5-fold by promoting the abiotic dissolution of basalt and upregulating microbial siderophore production to further accelerate weathering reactions. Moreover, EDDHA may alleviate potential Fe limitation of crops due to rising soil pH with EW over time. Initial cost-benefit analysis suggests potassium-EDDHA could lower EW-CDR costs by up to U.S. $77 t CO_2_ ha^–1^ to improve EW’s competitiveness relative to other CDR strategies.

## Abstract 2

Using microbial siderophores or commonly available synthetic chelators, iron chelation in soil accelerates enhanced rock weathering and increases its carbon dioxide removal efficiency by up to 2.5-fold.

## Introduction

Enhanced rock weathering (EW) is a promising scalable approach actively being researched for atmospheric carbon dioxide (CO_2_) removal (CDR).^1−3^ EW involves amendment of agricultural soils with crushed silicate rock, commonly the abundant volcanic rock basalt, to drive CDR by weathering reactions that release major cations (Mg^2+^, Ca^2+^, Na^+^, K^+^) and convert dissolved CO_2_ into soluble bicarbonate ions.^4−6^ EW provides agricultural cobenefits including improved crop production and enhanced soil fertility by ameliorating soil acidification, and increasing the availability of key plant macro- and micronutrients released from basalt grains by EW.^1,2,4,5^ Concerns over possible trace metal accumulation in soils have been raised^7^ but agricultural EW trials in the U.S. show no evidence of trace metal accumulation either in soils (pore water, exchange sites) or grains over four years of treatment.^1^ In response to the growing scientific evidence base, EW is scaling rapidly through worldwide expansion of commercial activities with significant purchases of the resulting carbon removal credits on the voluntary carbon markets (e.g., Microsoft, Stripe).^4,8^

Here, we focus on the manipulation of chelator activity in soil as a potential route to increasing the efficiency of EW and its resulting carbon removal. In addition, soil chelators could reduce potential Fe limitation on crop production^9−11^ caused by rising pH during EW. Iron is essential for metabolism including for protein cofactors^12^ and cytochromes involved in the electron transport chains for cellular respiration.^13^ Siderophores are high-affinity, highly selective iron (Fe) chelators produced by plants and microbes (bacteria and fungi) to counter the low biological availability of Fe and enhance the biological mass transfer of Fe into cells^14^ (Table 1). The oxic conditions of most soils render Fe biologically unavailable as most Fe is found as oxyhydroxides that are sparingly soluble across a range of soil redox and pH conditions.^15−18^ Fe(III)-siderophore complexes have orders of magnitude higher stability than those of other naturally occurring ligands such as organic acids (Table 1). These differences in stability mean that siderophores compete more efficiently for Fe binding than the hydroxo (OH^–^) group and other organic chelates over a larger pH range.^19^ Additionally, siderophores remain active in soils longer than low molecular weight organic acid anions, which are quickly metabolized by soil microbes.^20,21^ Siderophores are also typically recycled for further use without biochemical degradation.^22^

**Table 1:**
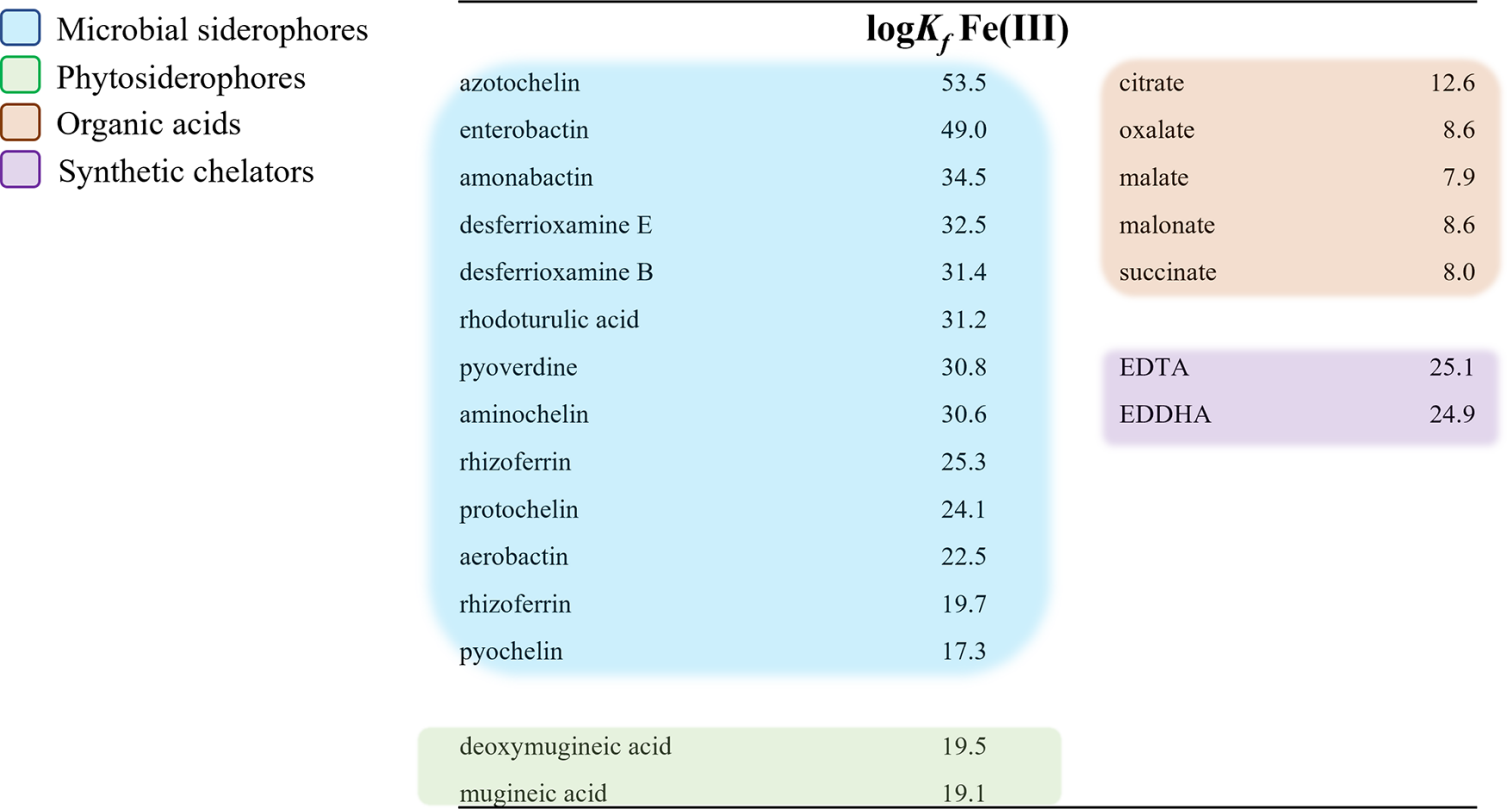
Stability Constants (log *K*_*f*_) for Ferric Iron of Several Natural and Synthetic Chelating Agents

Laboratory studies have established that siderophores drive the dissolution of a suite of Fe-bearing primary silicate minerals^23^ and secondary clay silicates^24−28^ by surface complexation. This accelerates the rate-limiting step of metal-oxide bond-breaking at the mineral-solution interface and increases the solubility of Fe(III) by solution complexation of dissolution reaction products.^29^ Microbial siderophores are therefore likely to play an active role in promoting EW with basalt, but this possibility remains to be investigated. We therefore evaluate this hypothesis with detailed “multiomics” analyses of the microbiomes sampled from basalt grain surfaces and soils in our ongoing EW field trials at the Energy Farm, Illinois, in the US Corn Belt.^2^

We next undertook proof-of-principle laboratory dissolution studies of basalt grains to assess the effectiveness of different classes of siderophores identified by our omics analysis. These experiments studied element release rates from a mineralogically well-characterized ground basalt feedstock (Table S1) composed of a mixture of Fe-bearing and other silicate minerals.^30^ Prior dissolution experiments with siderophores assessed their effects on individual Fe-bearing minerals^31^ or amorphous basaltic glass.^32^ These studies have shown that, e.g., low concentrations (e.g., 75 μM) of siderophores promote the release of Fe (50-fold), silicon (Si, 6-fold), and magnesium (Mg, 5-fold) from the Fe-bearing silicate olivine, a mineral constituent of basalt, relative to reaction with ligand-free solutions.^23^ Similarly, siderophore concentrations of 24 and 120 μM increased the dissolution of Fe (4–10 fold, respectively), Al (3–7 fold), and Si (1.7–2.2-fold) from hornblende.^33,34^ Thus, dissolution at low (micromolar) concentrations of microbial siderophores releases major elements other than iron to promote the dissolution of silicate minerals. However, the release of other elements from minerals by siderophores, and other strong chelating agents, depends on the mineral chemistry of the crystal lattice, with greater dissolution rates from Fe-rich minerals (e.g., olivine,^23,35^ iron-rich basaltic glass^36^) than their Fe-free counterparts (e.g., diopside,^37^ iron-free basaltic glass^36^). Given basalt deposits contain a sizable mass fraction of Fe-bearing ferromagnesian minerals including olivine, amphibole, and multiple pyroxene minerals (e.g., hedenbergite, ferrosilite, augite, pigeonite), with an average Fe content in basalts of 5–12 wt %, basalt rocks are likely susceptible to accelerated dissolution by chelators.

Building on these findings, we develop a potential biotechnology pathway for accelerating EW using a low-cost synthetic Fe-chelator to purposefully create Fe-deficient conditions for soil microbes and upregulate their siderophore production. The proposed pathway involves the application of the potassium form of the commercial synthetic chelate, ethylenediamine-*N*,*N*′-bis((2-hydroxyphenyl)acetic acid) (abbreviated EDDHA), commonly used in agriculture in prebound Fe-form to improve the yield of major crop species under Fe-deficient conditions,^38−40^ to basalt-amended soils. By scavenging readily available Fe(III) in soil and locking it into a form unavailable for direct microbial uptake,^41^ we hypothesize that iron-free potassium-EDDHA (K-EDDHA) stimulates microbial siderophore production and accelerates mineral dissolution. We also hypothesize that K-EDDHA forms surface complexes on basalt that additionally increase abiotic EW and Fe(III) release preventing back reaction by solution complex formation.^23^ Under these conditions, plants maintain the availability of Fe(III) derived from basalt in the presence of EDDHA via iron reductase at the root–soil interface.^42^ The previously complexed Fe(III) is liberated for bioutilization upon reduction to the Fe(II) form and is readily transported across the plant plasma membrane of root cells^42^ thus helping to alleviate potential Fe-limitation issues with rising soil pH and EW.

We evaluate these pathways to increasing EW and CDR with basalt using a series of incubation studies *in vitro* and with soils from U.S. EW trials across a range of EDDHA concentrations. These experiments assess the effects of EDDHA on (1) direct abiotic acceleration of basalt mineral dissolution and CDR potential and (2) stimulation of microbial siderophore production to further increase EW rates biotically. Finally, we discuss the broader implications of our work for amplifying CDR rates with EW on farmland and reducing costs.

## Materials and Methods

### Soil and Basalt Bag Collection for Omics Analyses

The weathered basalt bags and soils were collected from the long-term EW trials at the Energy Farm maintained by the University of Illinois at Urbana–Champaign and located in Urbana, Illinois (latitude: 40.064612, longitude: −88.196112). Polyethylene mesh bags (30 μm pore diameter) containing 4 g of fresh unweathered 63–90 μm basalt grains were heat-sealed (mesh dimensions: folded 4.0 × 4.0 cm^2^ triangles with the hypotenuse being 5.7 cm) and placed in the topsoil (0–10 cm depth) in the spring of 2021. After one and a half years of weathering in control soils (not previously treated with basalt) in fields rotationally planted with maize (*Zea mays* L. – 2021) and soybean (*Glycine max* L. – 2022), basalt bags and the soil surrounding them were collected in the summer of 2022 and immediately flash-frozen and stored on dry ice. Upon return to the laboratory, samples were stored at −20 °C for up to 1 year. The mesh bags are placed in soil to provide an experimental system for the *in situ* enrichment of basalt specialist microbes, allowing easy retrieval and subsequent analyses of microbial communities and basalt chemistry.^43^ They provide locales of high basalt/low soil effects naturally occurring in field trials (e.g., top-dressed application of crushed basalt or localized patches of basalt in tilled fields).

### Extraction of Nucleic Acids

Total DNA was extracted from weathered basalt samples (*n* = 20) and soils (*n* = 4) using the MagMAX Microbiome Ultra Nucleic Acid Isolation Kit (Thermo Fisher) and the instructions therein. The total RNA was extracted from the same samples with inputs of 1 g of soil and 1 g of weathered basalt using a modified method utilizing both the RNeasy PowerSoil Total RNA Kit (Qiagen) and MagMAX Plant RNA Isolation Kit (Thermo Fisher) protocols. Initially, soil or weathered basalt samples placed in 2 mL Eppendorf tubes were treated with 250 mL of beads solution followed by 25 μL of SR1 solution, 80 μL of SR2 solution, and 350 μL of phenol/chloroform/isoamyl alcohol. Samples were mixed using a GenoGrinder for three cycles each 5 min at 1250 rpm, with a rest time of 2 min in between cycles. After this, the samples were centrifuged at 2500*g* for 10 min, and supernatant (∼200 μL) was transferred to a new tube. To the supernatants, a volume of 150 μL of SR3 solution was added, and the samples were incubated for 10 min at 4 °C. Samples were then centrifuged for 10 min at 2500*g*, and the supernatant was transferred to a new tube. Next, the supernatants were treated following the RNA beads binding protocol from the MagMAX Plant RNA Isolation Kit (Thermo Fisher). Briefly, 10 μL of beads and 150 μL of absolute ethanol were added to each of the 200 μL supernatants for bead binding. The beads were washed with buffer wash once. Next, the supernatant was removed, and the beads were treated with 200 μL of DNase I mixture and incubated at 37 °C for 10 min. Samples were cooled to room temperature, and then 150 μL of rebinding buffer and 400 μL of absolute ethanol were added. After the beads were bound for 5 min, they were washed with washing buffer twice. Finally, the total RNA was eluted with 55 μL of RNAase-free H_2_O. The resulting RNA samples were purified using the OneStep PCR Inhibitor Removal kit (Zymo) and analyzed quantitatively and qualitatively on a Qubit and Agilent Bioanalyzer instruments.

### Shotgun Metagenomic and RNA-seq mRNA Library Preparation

Quality checks of the total DNA extracts revealed intact genomic DNA of high molecular weight in all samples without traces of degradation with an average concentration of 684 ng DNA g^–1^ weathered basalt (*n* = 16) and 4212 ng DNA g^–1^ soil (*n* = 4). The shotgun metagenomic libraries were prepared using the Illumina DNA Prep kit (previously Nextera DNA Flex kit) following the manufacturer’s instructions. The integrity check of the RNA samples was performed using the Agilent Bioanalyzer. Analyses revealed prominent bands associated with the small ribosomal and large ribosomal subunit rRNA indicative of reasonably high RNA integrity (mean RQN = 6.0 for weathered basalt RNA and mean RQN = 8.1 for soil RNA) for all but one highly degraded sample. The average yield for total RNA extractions was 22 ng RNA g^–1^ weathered basalt and 153 ng RNA g^–1^ soil. These total RNA extracts (*n* = 15 from weathered basalt and *n* = 4 from soil) were enriched for mRNA by rRNA depletion using the Illumina RiboZero Bacteria kit and RiboZero Yeast kit in a mixture with proportions of 80%:20%, respectively. The resulting mRNA-enriched libraries were then converted into cDNA libraries using the Illumina TruSeq Stranded mRNA kit and its associated protocols. MiSeq titration (26 nt + index) protocol was utilized for the final quantification of prepared libraries before their sequencing on one S4 flowcell of the Illumina NovaSeq 6000 system. Nucleic acid extraction, library preparation, and sequencing were carried out at the Roy J. Carver Biotechnology Center at the University of Illinois at Urbana–Champaign, US.

### Annotation of Siderophore Biosynthesis Genes within Shotgun Metagenomes and Metatranscriptomes

After initial in-house removal of adapter and index sequences, the cleaned sequencing R1 reads from each metagenome and metatranscriptome were uploaded to the Galaxy Europe server (https://usegalaxy.eu/)^44^ in FASTQ format and were then converted to FASTA. Each metagenome was functionally annotated against the IRcyc-A protein database^45^ using Diamond^46^ in its blastx mode (running parameters: *E*-value cutoff = 0.001, max number of hits = 1, BLAST tabular output containing query length in base pairs and subject length in amino acids) similar to previously utilized methods for Fe cycling gene annotation.^43^ IRcyc-A^45^ is a curated protein database for the detection and functional annotation of iron cycling genes and whole-microbiome taxonomy (based on the universal single copy gene *rpoA*(47) encoding the DNA-directed RNA polymerase α subunit) within omics designed to limit false-positive hits by filtering them off through matching to SwissProt^48^ protein sequences (general filter) and gene families paralogous to the iron cycle gene orthologues (specific filter). Hits matching target siderophore genes were then selected from the Diamond output by using the Select (Grep1) tool in the Galaxy.

The selected hits were downloaded and compiled for all 20 metagenomic and 19 metatranscriptomic samples at an *E*-value cutoff of 1 × 10^–5^. To account for intrinsic differences in gene length, each read was normalized for length by dividing its length (in base pairs) by the length of its matched protein subject (multiplied by three to convert from amino acids to base pairs). The total count of each gene orthologue (e.g., *desB*) within a metagenome or metatranscriptome was the sum of all length-normalized reads mapping to it (equivalent to the number of full-length copies mapping to it). Gene orthologues counts were then divided by the number of full-length copies of *rpoA* (universally present as a single copy in bacterial genomes^47^) to account for differences in sequencing depth among libraries. Sequencing reads mapping to siderophore biosynthesis genes in the IRcyc-A database (*E*-value cutoff of 1 × 10^–5^) were selected from raw FASTA files based on their sequence IDs and taxonomically annotated against the RefSeq database (version downloaded 12/2021) using Diamond.

### Harvesting Siderophores for *In Vitro* Dissolution of Basalt

Desferrioxamine B mesylate sodium salt (95% purity) was purchased commercially from Merck Sigma Aldrich. The remaining siderophores were obtained from growing selected bacteria under laboratory conditions. The selected bacteria included the *Burkholderia thailandensis* E264 wild-type (obtained as a freeze-dried culture from the Manoil Lab collection, University of Washington) and *Pseudomonas fluorescens* WDCM 00115 (purchased as vitroids from Merck Sigma-Aldrich). *B. thailandensis* E264 contains the genes necessary to produce the hydroxamate malleobactin, typically as a primary siderophore and pyochelin, usually as an accessory siderophore under iron-deficient conditions.^49^ Similarly, *P. fluorescens* is known to produce the mixed ligand siderophore pyoverdine as a primary siderophore and pyochelin as an accessory siderophore.^25^

Bacterial cultures were first grown as 10% tryptone soya broth prestarter cultures. Briefly, 25 μL of defrosted glycerol stocks or a single Vitroid discs were placed in sterile 50 mL tubes containing 20 mL of the 10% (w/v) tryptone soya broth medium under aseptic conditions. These were incubated for 2 days in the dark at 30 °C and 250 rpm. Liquid aliquots of the prestarter cultures were used to inoculate the specially designed low cation modified minimal medium (LCM^3^) with added iron, Fe + LCM^3^ medium to create starter cultures. The LCM^3^ medium was based on the MM9 minimal medium^50−52^ with modifications in the concentration of major cations to be able to reliably assess rock weathering rates. For 1 L of the Fe + LCM^3^ medium, the following were mixed: 0.8 L autoclaved buffer solution, 70 mL of the autoclaved salt stock solution, 30 mL of the filter-sterilized 10% (w/v) deferrated casamino acids + tryptophan, 50 mL of filter-sterilized deferrated succinic acid stock, 1 mL ammonium ferric citrate stock solution, and 49 mL ultrapure water to bring final volume to 1 L.

The buffer solution was made by adding to 0.750 L: 30.24 g of 2,2′-piperazine-1,4-diylbisethanesulfonic acid (PIPES) (C_8_H_18_N_2_O_6_S_2_) buffer, 0.3 g of KH_2_PO_4_, 0.5 g of NaCl, and 1 g of NH_4_Cl. The pH was adjusted to 6.8 using 50% KOH solution. Finally, ultrapure water was added to make the final volume of 0.8 L and the solution was autoclaved under pressure at 121 °C for 15 min.

The salt stock solution was made by adding the following to 1 L of ultrapure water: 0.8804 g of MgSO_4_·7H_2_O, 0.0786 g of CaCl_2_, 0.0167 g of MnSO_4_·H_2_O, 0.0200 g of H_3_BO_3_, 0.0171 g of ZnSO_4_·7H_2_O, and 0.0143 g of Na_2_MoO_4_·2H_2_O. Finally, 100 μL of CuSO_4_·5H_2_O stock (0.0029 g in 0.5 mL of ultrapure water) was pipetted into the 1 L mixture. The pH of the salt stock solution was adjusted to pH 6.8 using 50% KOH and the solution was autoclaved.

The 10% (w/v) deferrated casamino acids + tryptophan solution was made by dissolving 20 g of casamino acids and 0.3333 g tryptophan into 200 mL ultrapure water and adjusting to pH 6.8 with 50% KOH. For deferration, 200 mL 3% (w/v) 8-hydroxyquinoline (C_9_H_7_NO) in chloroform (CHCl_3_) were prepared by mixing 6 g of the chelating 8-hydroxyquinoline with 200 mL chloroform in the fume cupboard.^41^ One volume of the 3% (w/v) 8-hydroxyquinoline in chloroform solution was mixed with one volume of 10% casamino acids, and the mixture was vigorously hand-shaken for 1–2 min and allowed to deferrate for 48 h at 4 °C. Phases were separated, and the aqueous phase (containing the casamino acids) was collected and cleaned with chloroform until the chloroform was clear. The 200 mL deferrated casamino acid solution was split into 40 mL aliquots placed in five 50 mL tubes which were centrifuged at 4700*g* for 5 min and supernatants were filter-sterilized through 0.2 μm syringe filter into a sterile tube under sterile conditions (laminar flow).

The deferrated succinic acid stock solution was made by dissolving 8 g of succinic acid in 200 mL of ultrapure water. The pH was adjusted to pH 6.8 using 50% KOH. Equal volumes of 3% (w/v) 8-hydroxyquinoline in chloroform and succinate stock were mixed to deferrate as described above. Finally, the 200 mL deferrated succinic acid stock solution was split into 40 mL aliquots placed in five 50 mL tubes, each centrifuged at 4700*g* for 5 min and filter-sterilized through 0.2 μm syringe filter into a sterile tube under sterile conditions (laminar flow).

The ammonium ferric citrate stock solution is only added to Fe + LCM^3^ and omitted when preparing the iron-deficient Fe-LCM^3^ medium. To make the solution, 0.0588 g of ammonium ferric citrate is added to 2 mL of ultrapure water and dissolved by pipetting in and out. The mixture is filter-sterilized using 0.2 μm syringe filters into a sterile Eppendorf tube under aseptic conditions.

After 2 days of growth, the Fe + LCM^3^ starter bacterial cultures were then transferred for growth in the iron-deficient conditions promoting siderophore biosynthesis of the Fe-LCM^3^ medium. This was achieved by appropriately diluting starter cultures with Fe-LCM^3^ to obtain 1.2 mL cultures at an OD_600_ = 0.6. These were then centrifuged at 8400*g* × 1 min to pellet the cells. The supernatant was discarded, and the pellet was resuspended in 1.2 mL of Fe-LCM^3^ medium. This step was repeated a total of three times to remove any traces of iron-containing Fe + LCM^3^ medium and replace it completely with the iron-deficient Fe-LCM^3^ medium. Finally, 180 μL aliquots of the resulting cell suspensions in Fe-LCM^3^ were added to 30 mL Fe-LCM^3^ in 50 mL tubes—in five replicates per bacterial species. These were grown for 3 days in the dark at 30 °C and 250 rpm. Cell-free siderophore solutions were harvested after 3 days of growth. To do so, each culture tube was centrifuged at 8400*g* for 1 min and supernatants were filter-sterilized using a 0.2 μm syringe filter into sterile tubes under aseptic conditions. The resulting siderophore solutions were kept at 4 °C until ready to measure their siderophore concentration and then utilized for the basalt weathering experiments.

### Quantification of Siderophores in Cell-Free Suspensions

The siderophores were quantified using a modified^51^ version of the Chrome Azurol S (CAS) assay.^50^ Briefly, 1.5 mL of 1 mM FeCl_3_·6H_2_O in 10 mM HCl was mixed with 7.5 mL of the 2 mM CAS (0.3023 g Chrome Azurol S (C_23_H_13_C_l2_Na_3_O_9_S) to 250 mL ultrapure water). The resulting mixture was added to 25 mL of 2.4 mM hexadecyltrimethylammonium bromide (HDTMA) solution (0.0219 g of HDTMA (C_21_H_42_NBr) in 25 mL of ultrapure water dissolved by magnetic stirring over low heat). Finally, 50 mL of 2-(*N*-morpholino)ethanesulfonic acid (MES) buffer solution (9.76 g MES (C_6_H_13_NO_4_S) in 50 mL ultrapure water adjusted to pH 5.6 using 50% KOH) were added to the mixture and volume was made up to the 100 mL mark using ultrapure water. The obtained CAS dye reagent was split into 5 mL aliquots and stored at 4 °C before use. Immediately before being used for the measurement of siderophore equivalent concentrations in solution, 15 μL of sulfosalicylic acid stock solution (0.2543 g 5-sulfosalicylic acid dihydrate (C_7_H_10_O_8_S) to 5 mL ultrapure water) were added to each 5 mL CAS dye reagent aliquot. To perform the spectrophotometric CAS assay, 200 μL of standard, neat cell-free culture filtrate or sixfold diluted culture filtrate were mixed with 200 μL of activated CAS assay dye solution in an ultramicro cuvette. These were incubated at room temperature for 2^1^/_2_ h. After that, the absorbance was measured at a 630 nm wavelength. For the standard curve, a range of desferrioxamine B mesylate standards (0, 1, 3, 5, 7.5, 12.5 μM—low range and 15, 20, 25, 30 μM—high range) were prepared in fresh Fe-LCM^3^ medium.

### *In Vitro* Dissolution of Basalt Experiments with Siderophores

Hillhouse basalt rock^30^ was sieved to 53–75 μm grain diameter and ∼12 g of these grains were placed in a 50 mL tube and acid-washed with 30 mL 0.01 M HCl by vigorous shaking to remove exchangeable ions and nanometer-scale dust particles resulting from the rock grinding process. The acid was discarded, and the basalt was further washed with ultrapure water a total of six times to remove any traces of the acid. The purified stripped basalt was then dried for 48 h at 80 °C. A total of 0.05 g dried basalt grains were added to 25 mL acid-washed universal glass bottles with autoclavable white plastic lids. The basalt and its containing tube were autoclaved for one cycle. Each of the basalt samples was weathered under three sequential phases, each of which lasted for 90 h.

In the first phase, basalt was reacted with 10 mL of filter-sterilized CO_2_-saturated ultrapure water. At the end of the 90 h of the first phase, the reacted solution was pipetted out into a fresh tube without taking any basalt grains from the glass tube. Grains were prewashed (thus containing no fines below the specified range of 53–75 μm grain diameter), meaning there was no need for centrifugation as this grain fraction settles quickly (within 10 s). The reacted solution was obtained by slowly pipetting from the side opposite to where the grain pellet formed. In the second phase, the basalt was reacted with 10 mL of filter-sterilized Fe-LCM^3^ medium containing either siderophores [1, 5, 10, 20, and 40 μM desferrioxamine B mesylate (DF) or 1–40 μM DF equivalents as measured by the CAS assay of malleobactin/pyochelin mixture or pyoverdine/pyochelin mixture] or disodium citrate (citrate content of 40–300 μM), also including a control treatment (only fresh Fe-LCM^3^ medium without added chelators). After 90 h, the reacted solution was pipetted out into a fresh tube as described above. Lastly in the final phase, each of the basalt samples was again reacted with 10 mL of filter-sterilized CO_2_-saturated ultrapure water. After 90 h, the reacted solution was pipetted out into a fresh tube as described above.

All of the preparatory operations were carried out in the laminar flow cabinet under aseptic conditions to secure abiotic cell-free weathering of the basalt grains. The 90 h weathering period was carried out in the dark at 25 °C and on an orbital shaker set at 201 rpm. The CO_2_-saturated ultrapure water was obtained by using a pressurized food-grade 100% CO_2_ gas cylinder within the SodaStream system and their specified bottle. To limit CO_2_ degassing, the bottle was placed in a bucket filled with ice, and the CO_2_-saturated water was syringe-filtered through sterile 0.2 μm filters in portions into sterile 50 mL tubes also placed on ice before the solution was added to the basalt samples. All of the weathering reactions were carried out in replicates of four (*n* = 4) for each treatment. The elemental composition of the weathering solution from each phase was determined by inductively coupled plasma mass spectrometry (ICP-MS). To prepare samples for ICP-MS analysis, each reaction solution was diluted five times, filtered, and reacted to a final concentration of 2% nitric acid content and left at 4 °C overnight. If organic precipitate had formed (common to the solutions from the phase 2 ligand weathering) the samples were centrifuged for 5 min at 4700*g* and syringe-filtered one final time using 0.45 μm filters into fresh 15 mL tubes and sent together with blank solutions from each phase for ICP-MS analyses at the Environmental Lab, University of Nottingham, U.K. To estimate the rates of dissolution, the blanks were deducted from their respective samples.

### *In Vitro* Basalt Dissolution Experiments with EDDHA or Desferrioxamine

For this experiment, 0.07 g aliquots of washed 53–75 μm grain diameter Hillhouse basalt feedstock were placed in 50 mL flat-bottomed sample cups, and 14 mL of chelator-free or chelator-containing 0.001 M KCl solution were added. The chelators used were desferrioxamine B mesylate sodium salt and ethylenediamine-*N*,*N*′-bis(2-hydroxyphenyl)acetic acid (EDDHA, C_18_H_20_N_2_O_6_) and their concentrations were adjusted for the following three different tests −40, 375, and 730 μM using a 1200 μM stock solution for each chelator neutralized to pH 7.0 using KOH. Sample cups were then amended with parafilm on top to limit evaporation but allow for equilibration with atmospheric CO_2_ levels. The experimental replicates (*n* = 4 for each concentration for each chelator) were placed for 90 h dissolution phase at 150 rpm, 25 °C in the dark.

Following the end of the incubation period, 9 mL aliquots of the solution from each reacted basalt sample were placed in glass vials and analyzed for their total dissolved inorganic carbon (DIC) concentration using a Shimadzu TOC-L machine precalibrated with freshly prepared sodium bicarbonate standards (0, 0.5, 1, 2, 4, 8, 16, 32 mg C L^–1^). The remaining 5 mL were syringe-filtered using 0.45 μm filters and were then used for pH determination. Subsequently, samples were diluted fivefold and acidified to 2% HNO_3_ and sent for ICP-MS analysis to quantify the elements dissolved from basalt during the dissolution. Concentrations of HCO_3_^–^ were determined using established equilibrium constants for CO_2_ speciation in water and the measured pH—these were used to derive the % of dissolved CO_2_ in the bicarbonate form. These were converted to concentrations by multiplying the estimated %HCO_3_^–^ by the total DIC measured.

### Soil Microcosm Incubations with Potassium-EDDHA

The soil used for the incubations was obtained fresh from the untreated block 5 within the long-term EW field trials at the Energy Farm site. Fresh field soil was sieved to <2 mm and stored before the experimental setup at 4 °C. For basalt-treated samples, 1 g of moist (10% w/w) soil was mixed with 0.25 g of washed and dried 53–75 μm Hillhouse basalt grains and added to a sterile 15 mL tube. For each of the untreated soil samples, 1.25 g of soil was added to a sterile 15 mL tube. Taking into account the initial soil moisture levels, each of the samples was brought to soil moisture of 30% (w/w) by treating with an organic nutrient solution designed to provide labile organic C for the microbial community. The rationale for adding 20 mg g^–1^ was to replicate typical formulations of minimal nutrient media for microbial growth that supply 1–2% (w/w) carbon substrate. The solution contained potassium succinate with or without chelator to reach a final concentration of 0.02 g succinate g^–1^ soil or soil + basalt substrate in the treated sample. Depending on the four different chelator treatments used in the experiment −0, 100, 425, or 750 μM ethylenediamine-*N*,*N*′-bis((2-hydroxyphenyl)acetic acid) [EDDHA] concentrations in soil solution, the watering solution used to bring soil moisture to 30% also contained EDDHA at the necessary concentrations by adjusting the amount of added 1500 μM EDDHA stock solution. The 20 μM prebound Fe:desferrioxamine B complex used in the [425 μM EDDHA + 20 μM Fe:desferrioxamine] treatment was obtained by prereacting 100 μM desferrioxamine B mesylate stock with equal volumes of 100 μM FeCl_3_·6H_2_O in 10 mM HCl and bringing pH to 7.0 using 10% KOH. Once watered the tube screw caps were placed and the soil microcosms were incubated in the dark at 25 °C. The soil samples were destructively sampled in replicates of three (*n* = 3) per treatment at the beginning of the experiment as well as at 3, 10, and 20 days after the start of the experimental incubation. At each time point, 3 mL of ultrapure dH_2_O was added to each soil sample and samples were extracted sequentially as specified in Figure S6. After the water extract, samples were reconstituted with 12.5 mL of 1 M ammonium acetate (C_2_H_7_NO_2_) buffered at pH 7.0, and their exchangeable ion fraction was extracted (Figure S6). Syringe-filtered and appropriately diluted hot water extracts (filtered through 0.22 μm) and 1 M ammonium acetate extracts (filtered through 0.45 μm) were acidified to 2% HNO_3_ and sent for ICP-MS analyses to investigate the patterns in soil solution-available or exchangeable ions, respectively. Hot-water extraction of soils allows characterizing the effects of EW on soil chemistry in field trials.^1^ We used this approach for two reasons. First, using distilled water allows for extracting the soil with less change in its pH compared to other extractants (e.g., ammonium acetate is buffered at pH 7.0 or 8.5,^53^ Mechlich-III extraction solution is pH 2.5^53^). This is important for nutrients whose availability is pH-dependent (e.g., Fe,^54,55^ Mo,^56^ Cu, Zn, Si^57,58^ etc.). Because basalt-amended soils typically experience an increase in pH,^1,2,6^ the use of fixed pH-extractants is not suitable. Second, water is sufficiently mild to avoid weathering the basalt during the extraction procedure unlike other extractants (e.g., DTPA-based extraction,^53^ 0.5 N acetic acid-based extraction,^59^ etc.). The heating of the sample releases more nutrients from their organo-mineral form,^60^ mimicking microbial decomposition. Overall, this approach provides a means of determining, in as close approximation as possible, the soil nutrient status in EW trials and experiments.

Cold water extracts were filter-sterilized using 0.2 μm syringe filters and used in the *Arthrobacter* sp. JG-9 (now updated to *Mycobacterium flavescens* JG-9) bioassay to estimate free and adsorbed hydroxamate siderophores in soil.^61,62^ The *Arthrobacter* sp. JG-9 culture was acquired from the NCIMB collection under NCIMB 9471. The detailed growth protocol for *Arthrobacter* sp. JG-9 and more information about the bioassay can be found in Note S1, Supporting Information.

## Results and Discussion

### Multiomics Analyses Identify Microbial Siderophore Upregulation Response to Basalt

We analyzed the response of the belowground microbiome for *in situ* siderophore production by extracting both total DNA and RNA from reacted basalt rock grain samples (*n* = 16) weathered for 1^1^/_2_ year in the soil of a maize/soybean rotation in the U.S. EW Corn Belt and compared them to the community of the surrounding soil (*n* = 4; Figure S1). Performing high-throughput Next-generation sequencing of the resulting DNA and mRNA-enriched cDNA-RNaseq libraries, we obtained information on both the total relative abundance (metagenomics) as well as expression levels (metatranscriptomics) of key siderophore biosynthesis genes in the communities associated with weathered basalt and the surrounding soil.

**Figure 1:**
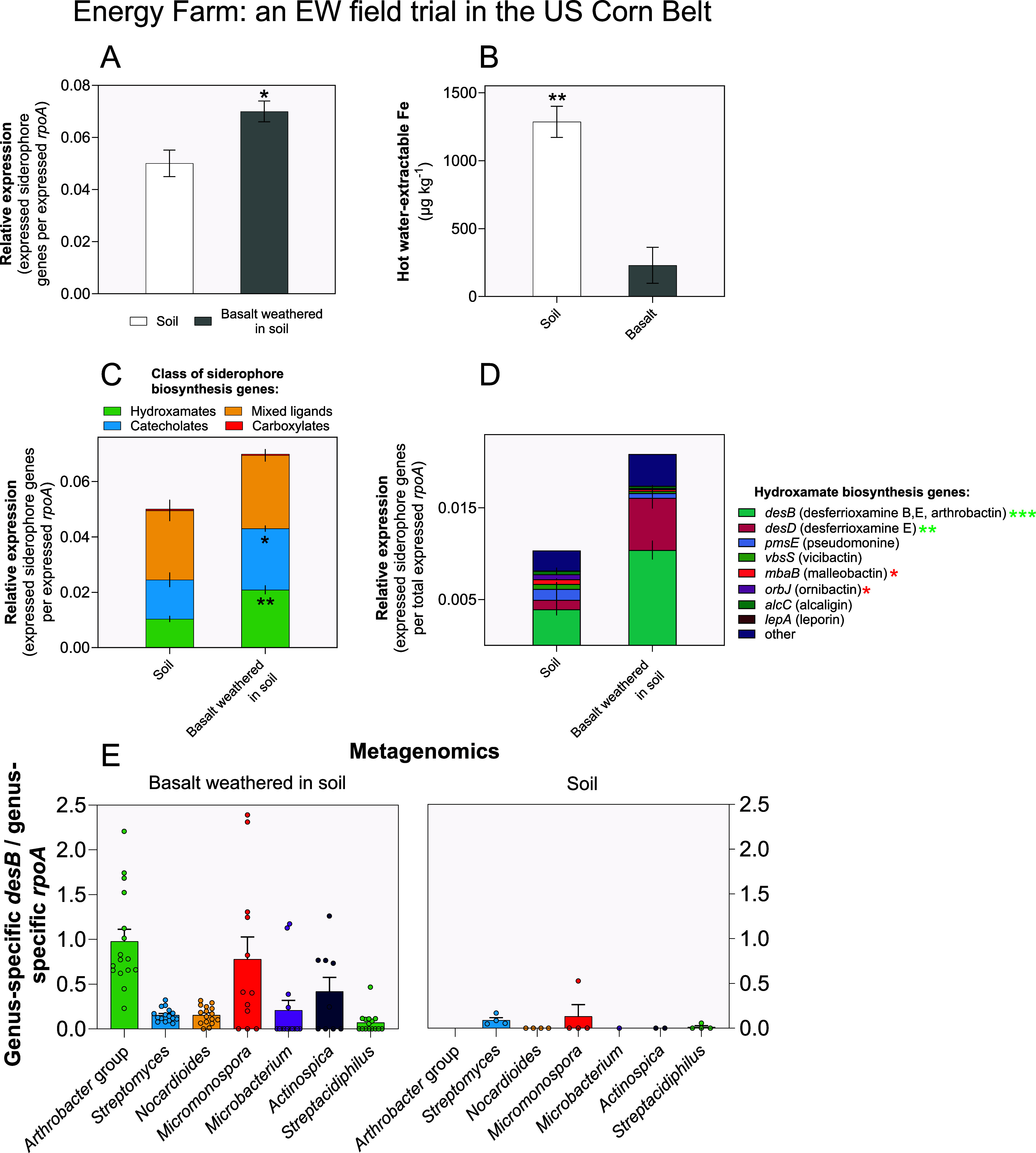
Omics analyses of microbial siderophore production during enhanced rock weathering (EW) trials in the U.S. Corn Belt agroecosystems. (A) Metatranscriptomic RNA-seq analyses show greater expression of siderophore biosynthesis genes in the in situ basalt microbiome than in the surrounding soil microbiome (two-tailed *t* test, *P < 0.05). (B) Hot water-soluble iron (Fe) levels are significantly lower in basalt than in the surrounding soil matrix (two-tailed *t* test, **P < 0.01). (C) Siderophore biosynthesis gene classification reveals significantly greater expression of genes involved in the production of hydroxamates and catecholates than other groups (two-tailed *t* test, **P < 0.01, * < 0.05). (D) Breakdown of hydroxamate biosynthesis genes into separate siderophore types shows that desferrioxamines and arthrobactin are among the most upregulated in the basalt microbiome. Green and red asterisks indicate significant increases or decreases, respectively, in basalt over control microbiomes (two-tailed *t* test, ***P < 0.001, ** < 0.01, * < 0.05). (E) Greater genus-specific ratios of *desB* to *rpoA* (a gene present as a single copy in microbial genomes) in the metagenomes of in situ basalt relative to soil indicate positive selection for microbes producing desferrioxamines/arthrobactin. This pattern is robust across key genera. *rpoA* = DNA-directed RNA polymerase subunit α. Error bars show the standard error of the mean (SEM). Replication for each substrate was as follows: *n* = 15 (metatranscriptomics) and *n* = 16 (metagenomics) replicates for soil-weathered basalt rock microbiomes, *n* = 4 replicates (both types of omics) for the surrounding soil microbiomes.

Our findings indicate that the microbiomes of weathered basalt grains exhibited a significant 40% increase in relative expression levels of siderophore biosynthetic genes (two-tailed *t* test, *P* < 0.05) compared to soil communities (Figure 1A). We show that at native pH, soil contains 3-fold higher levels of readily available Fe compared to basalt (two-tailed *t* test, *P* < 0.001; Figure 1B) due to being more acidic than basalt (soil pH = 5.6 compared to basalt pH = 8.1), which directly influences the availability of Fe.^18^ Our findings suggest that the upregulation of siderophore biosynthesis in basalt grain microbiomes is related to low Fe availability compared to that for microbes in bulk soil.

Functional classification of the stated siderophore genes into their respective siderophore biochemical classes indicated that the most responsive were those encoding the biosynthesis of the hydroxamate class of siderophores with over 2-fold greater expression (two-tailed *t* test, *P* < 0.01) in basalt grains compared to soil (Figure 1C). Similarly, genes encoding for the biosynthesis of the catecholate class of siderophores exhibited 76% higher relative expression levels (two-tailed *t* test, *P* < 0.05) in basalt than in soil (Figure 1C), with the classes of mixed ligand and carboxylate siderophores remaining largely indifferent in expression between rock and soil microbial communities.

Next, we undertook analyses of the specific gene orthologues encoding separate types of hydroxamates to assess the most responsive hydroxamate siderophore biosynthesis genes in basalt microbiomes. Our analyses reveal that the *desB* gene orthologues represented the highest expressed siderophore gene orthologous group, accounting for ∼50% of all expressed hydroxamate genes and up to 15% of all siderophore genes expressed (Figure 1D). The *desB* orthologous group genes encode the enzyme cadaverine hydroxylase^63^ implicated in the transformation of l-lysine to desferrioxamine B, desferrioxamine E, and arthrobactin siderophores.^64^ The *desB* and the desferrioxamine E synthetase gene *desD* groups exhibited significant 2.6-fold and 5.6-fold upregulation (two-tailed *t* tests, *P* < 0.001 and <0.01; Figure 1D), respectively, in basalt grain microbiomes relative to soil microbiomes suggesting that desferrioxamines and arthrobactin siderophores are key to microbial life on actively weathering basalt grains.

Taxonomic profiling of the main hydroxamate gene *desB* in soil and weathered basalt grain metagenomes against the RefSeq database^48^ confirms that the majority of *desB* genes belong to members of the Actinobacteriota (as are all listed genera in Figure 1E). This highlights that Actinobacteriota are key drivers of siderophore production in belowground systems and is consistent with culture-based findings showing a diverse array of siderophore-producing bacteria belonging to the phylum of Actinobacteriota (also commonly referred to as actinomycetes).^64−66^

To determine if the capacity to produce siderophores is selected for the basalt grain microbiome relative to that of soil, we calculated the ratio of *desB* to the single copy marker *rpoA* on a genus-specific basis using our shotgun metagenomics data (Figure 1E). A higher genus-specific *desB*/*rpoA* ratio would mean that a greater proportion of members of this genus carried the *desB* gene (more *desB* per genome), whereas a smaller *desB*/*rpoA* ratio would indicate that fewer members of the genus carried the gene (fewer *desB* per genome). Results (Figure 1E) indicate that a greater proportion of bacterial genomes carried the *desB* gene in basalt across all major genera tested relative to soil communities. Similarly, expression levels followed the same pattern (Figure S2). These findings suggest that basalt grain microbiomes are shaped by a positive selective pressure for the recruitment of siderophore-producing strains of the same genus and that siderophore biosynthesis is a fitness-associated trait for unlocking, extracting, and transporting nutrients.

### Proof-of-Concept: Versatile Siderophores Increase In Vitro Dissolution of Basalt

Our subsurface multiomics analyses implicate an active role for siderophores in basalt dissolution, but this requires confirmation with laboratory dissolution studies. We therefore undertook a set of time-dependent abiotic dissolutions at a circumneutral pH of 6.8 using a well-characterized basalt feedstock comprising Fe-containing silicate minerals, including olivine, augite, and biotite^30^ (Table S1).

**Figure 2:**
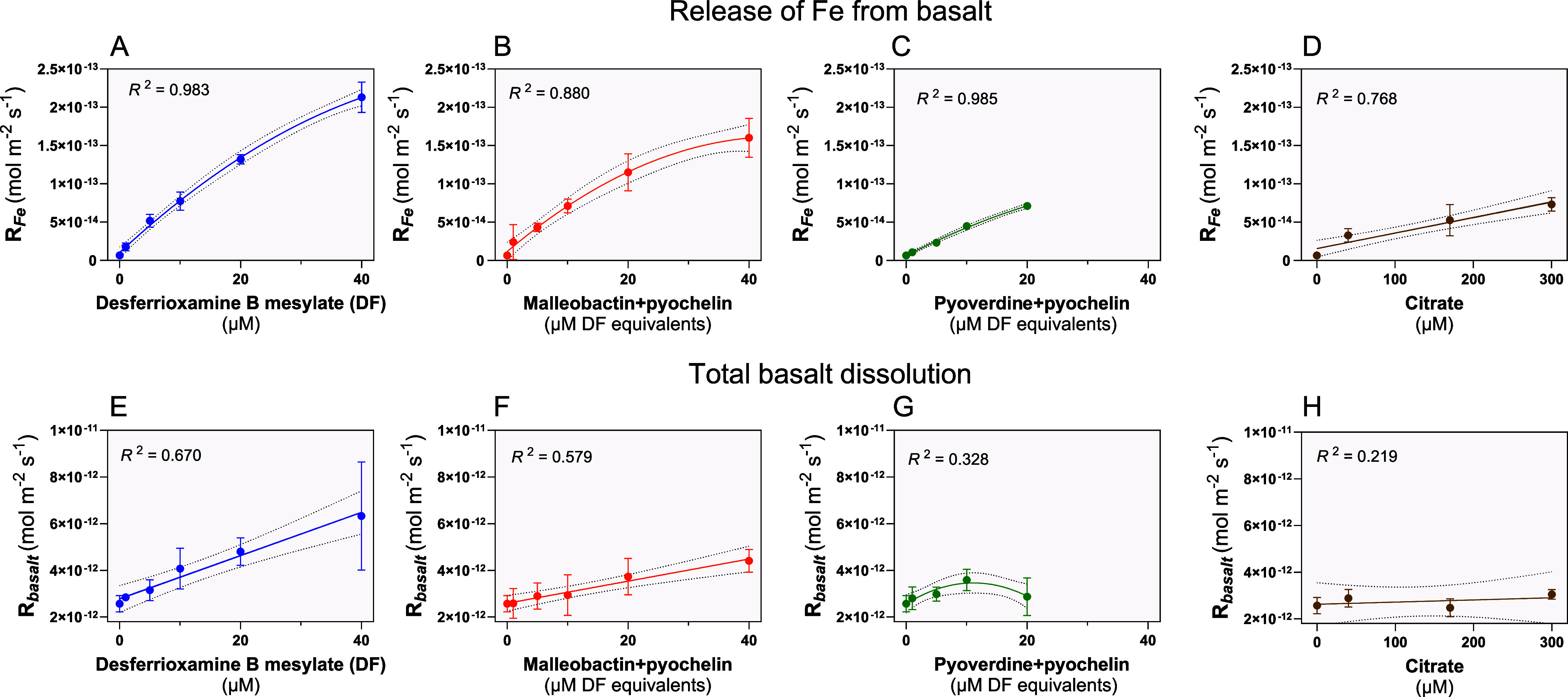
Diverse siderophores increase in vitro iron and basalt dissolution. (A) Weathering rate of iron (Fe) release (*R*_Fe_) from basalt in response to desferrioxamine B mesylate (DF) dissolved in a microbial medium (1–40 μM). (B) Release rate of Fe from basalt by weathering in response to cell-free supernatant from Fe-deficient *B. thailandensis* E264 culture containing malleobactin and pyochelin (1–40 μM DF equivalents). (C) Release rate of Fe from basalt by weathering in response cell-free supernatant from Fe-deficient *P. fluorescens* ATCC 13525 culture containing pyoverdine and pyochelin (1–20 μM DF equivalents). (D) Release rate of Fe from basalt by weathering in response to citrate dissolved in the microbial medium (40–300 μM citrate). (E–H) Rates of basalt weathering in response to the same conditions described in (A)–(D) *R*_basalt_ is calculated as the sum of *R*_Mg_, *R*_Ca_, *R*_Na_, *R*_Si_, *R*_Al_, *R*_Ti_, and *R*_Fe_. Error bars show SEM. Akaike’s Information Criterion (AICs) was used to compare quadratic vs linear model and the higher-ranking best-fit model was used. Replication for each concentration and each chelator in replicates of four, *n* = 4. The non-SI units “μM DF equivalents” are used since the concentration of unpurified siderophores is measured based on their activity in the CAS assay (see Materials and Methods) relative to a standard curve based on known amounts of desferrioxamine B mesylate (DF).

During the time-dependent dissolution of basalt, substantial increases in dissolved Fe were observed following the addition of a Fe chelator (siderophore or citrate) in a concentration-dependent manner (Figure 2A–D). At the same total concentrations, siderophores were substantially more effective at Fe solubilization than the organic ligand, citrate. For example, 20–40 μM siderophore concentrations resulted in 11–33-fold in Fe release from basalt grains (Figure 2A–C), compared with the 5-fold increase for citrate (40 μM, both relative to ligand-free control, Figure 2D).

Different types of siderophores associated with diverse microbes exhibited significant differences in their reactivity to dissolve Fe from basalt. For instance, at the same concentration of 20 μM, actinobacteria-produced desferrioxamine B had the highest dissolution rates (20-fold), followed by proteobacterial malleobactin (18-fold), with pseudomonad-generated pyoverdine being the least effective (11-fold; Figure 2A–C). These findings for the abiotic reaction are consistent with differences in the kinetic reactivity of surface-bound ligand to dissolve the mineral or steric hindrance of surface binding capacity caused by the increasing size of the siderophore molecules; *M*_r__desferrioxamine B_ ≤ *M*_r__malleobactin_ ≪ *M*_r__pyoverdine_.

Siderophore addition significantly increased basalt dissolution, as measured by accumulation in solution of the total elemental concentrations of Si, Ca, Mg, Na, Ti, Al, Fe, and Mn, with increasing added ligand concentration (Figure 2E–G). The element release followed similar patterns in relative Fe dissolution reactivity of the three siderophores. The scale of this increased weathering effect followed a significant linear pattern for siderophores, varying from 10 to 20% at low siderophore concentrations (1–5 μM) to 40–150% at high concentrations (except pyoverdine, which followed linear patterns only in the range 1–10 μM; Figure 2E–G). These findings suggest a kinetic mass-action effect of siderophores on mineral lattice stability and enhanced cation release. Thus, even at low concentrations typical in the soil environment (∼1.0 μM hydroxamates in soil solution, unadsorbed^67^), microbial siderophores could increase the weathering of basalt grains. In contrast, citrate failed to drive significant total basalt dissolution (Pearson correlation test, *P* > 0.10, Figure 2H), indicating that the natural concentration range of citrate found in soil (∼20 μM for mineral soil and 72 μM for organic horizons^68^) may stimulate Fe release (Figure 2D) but have negligible effects on total basalt dissolution (Figure 2H) at the circumneutral pH.

The limited effect of citrate maybe even more exacerbated in basalt-amended soil. In this situation, citrate could lose much of its Fe-complexing ability with the resulting pH increase effectively leading to it preferentially complexing with Ca^2+^ in soil solution; micromolar concentrations of citrate would be overwhelmed by the millimolar concentrations of Ca^2+^ in soil solution.^19,69^ Similarly, although oxalate and siderophores can interact synergistically *in vitro* to dissolve Fe-oxyhydroxide minerals,^70−72^ in neutral soil solutions with dissolved Ca^2+^ and Mg^2+^, oxalate, could also lose its Fe(III)-chelating properties.^19^

Our results showing nonlinear dependence of Fe release rate on solution ligand concentration are consistent with rate expressions derived from geochemical theory. Quadratic polynomial function fitted better than a simple linear relationship for siderophores (Akaike’s Information Criterion test) suggesting that saturation is reached at higher siderophore concentrations. Dissolution kinetic behavior is predicted to exhibit a first-order dependence on dissolved ligand concentration at low surface coverage and progressive transition to zero-order kinetics at increasing solution concentration of the reacting ligands. The transition to zero-order kinetics occurs due to adsorption saturation of dissolution-reactive ligand-binding sites on the mineral surfaces.^73^

Using desferrioxamine B mesylate at 20 μM as an example, we show that in the presence of siderophores Fe release increased by 20-fold, Al release by 41-fold, Ti release by 4.3-fold and Si release by ∼2-fold (two-tailed *t* tests, *P* < 0.001) (Figure S3). In addition, we detected significant increases in the total dissolved divalent cations, including a 2.4-fold increase in Ca at a concentration of 20 μM (two-tailed *t* test, *P* < 0.05).

### *In Vitro* Basalt Dissolution: Siderophores and Synthetic Chelators Increase CDR Rates

**Figure 3:**
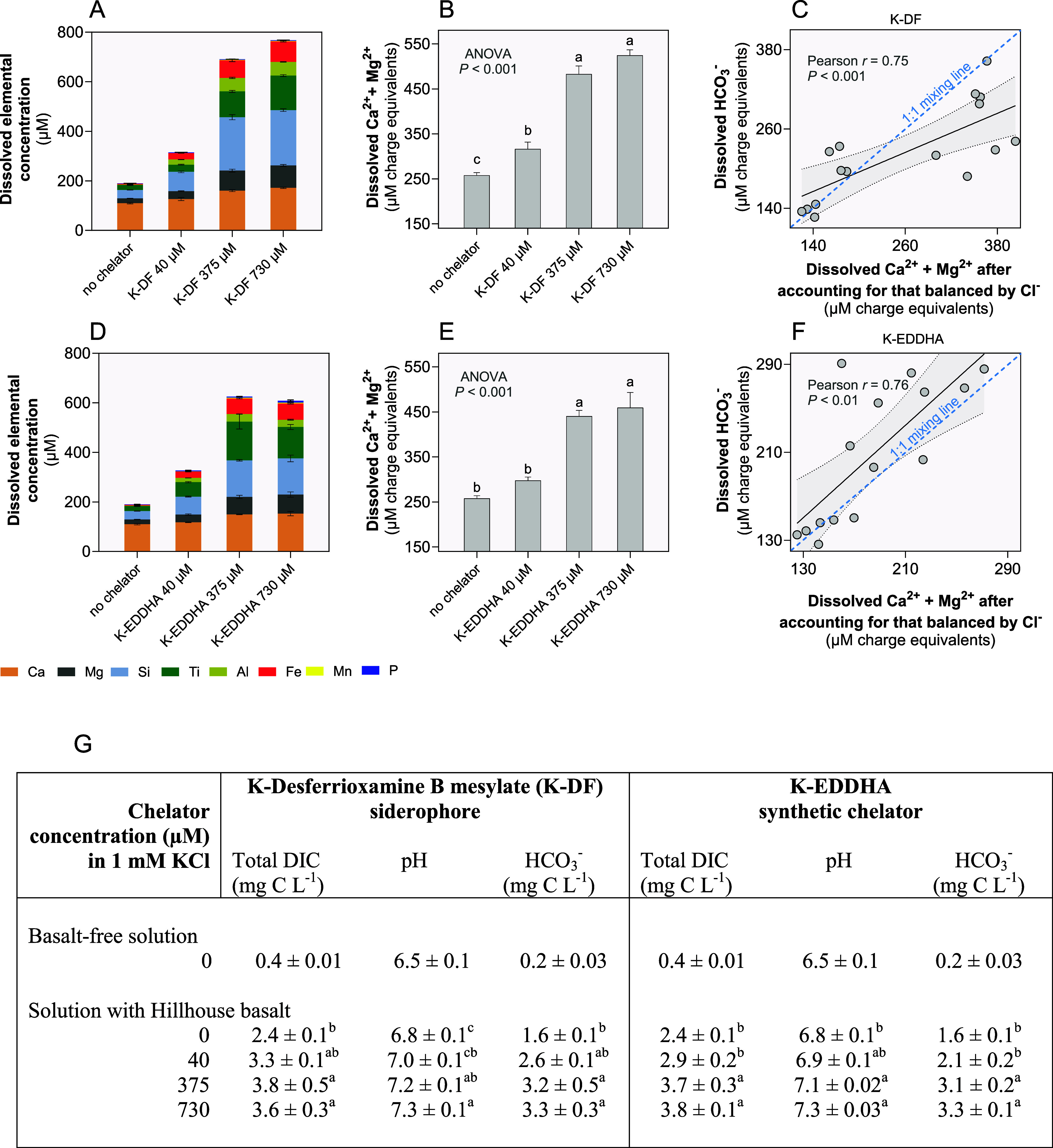
Effects of the high-affinity chelators potassium desferrioxamine B mesylate (K-DF) and potassium-EDDHA (K-EDDHA) on basalt dissolution, alkalinity, and CDR in vitro. (A) Dissolved concentration of elements from basalt over 90 h incubation at atmospheric *p*CO_2_ in response to varied K-DF concentrations. (B) Summed dissolved divalent base cations (Ca^2+^ + Mg^2+^) in response to varied K-DF concentrations. (C) Correlation between the sum of dissolved (Ca^2+^ + Mg^2+^) concentration and measured HCO_3_^–^ concentration in K-DF treatment. (D) Dissolved concentration of elements from basalt over 90 h incubation at atmospheric *p*CO_2_ in response to varied K-EDDHA concentrations. (E) Sum of the dissolved divalent base cations (Ca^2+^ + Mg^2+^) from basalt in response to varied K-EDDHA concentrations. (F) Correlation between dissolved the sum of dissolved (Ca^2+^ + Mg^2+^) and measured HCO_3_^–^ concentration in K-EDDHA treatment. (G) Tabulated measurements of solution DIC, pH, and HCO_3_^–^ over the basalt-free solution compared to solution with Hillhouse basalt dust added at varied chelator concentrations. Statistical tests performed include the Pearson correlation test and analysis of variance (ANOVA) tests with Benjamini–Hochberg multiple comparison correction. Different letters signify significantly different means. Values in the table show mean ± SEM. Replication for each concentration for each chelator in replicates of four, *n* = 4.

We directly assessed the potential of solutions of the potassium salt of siderophore desferrioxamine B mesylate and EDDHA, which form highly stable dissolved and surface complexes with metal ions, to increase basalt weathering and CDR. We show that in the presence of such strong chelators the pH of the weathering reactions increased with increasing chelator concentrations (Figure 3G). These results are consistent with the enhanced consumption of H^+^ during chelator-promoted weathering through the chemical dissolution of the oxide mineral framework of basaltic minerals to form dissolved orthosilicic acid. These results were consistent for both chelators investigated. The highest chelator concentration (730 μM) showed an ∼2-fold increase in the HCO_3_^–^ concentration in weathering solutions with either desferrioxamine B mesylate or EDDHA relative to chelator-free controls (Figure 3G). Similar to our previous experiment with diverse siderophores at lower concentrations (1–40 μM; Figure S3), the dissolved concentrations of metals and silicon across the high range of chelator concentrations (40–730 μM) tested indicate enhanced basalt dissolution with increasing mass transfer of Fe, Al, Ti, Si, Mg, and Ca to solution for both ligands (Figure 3A,D). Both the siderophore and EDDHA significantly increased the release of divalent cations from basalt relative to the chelator-free control (Figure 3B,E). The summed concentration of divalent Ca^2+^ and Mg^2+^ base cations released during weathering correlated strongly with the concentration of HCO_3_^–^ as calculated from analytically determined −log{H^+^} and dissolved inorganic carbon (DIC) concentrations (Figure 3C,F). These results indicate that base cation concentrations produced from basalt dissolution in response to strong chelating agents can be used as reliable proxies for increases in the potential CDR.

At the highest concentration of 730 μM, the potassium salts of the siderophore desferrioxamine B promoted the dissolution of multiple elements from basalt with the release pattern calculated in folds-over the release observed in the chelator-free control following the order
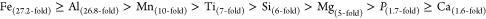
Similar was the progressive sequence of elemental release from basalt mediated by the potassium salt of EDDHA
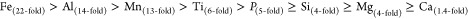


However, the pattern of phosphorus and aluminum release differed from the above elements. K-EDDHA promoted the dissolution of phosphorus from basalt to a significantly greater extent than the K-desferrioxamine B siderophore (Figures S4 and 3A,D). Also, EDDHA exhibited a significantly lower preferential release of Al than the siderophore (Figure 3A,D).

These patterns of elemental release are broadly consistent with the three different siderophore mixtures investigated at lower concentrations (Figure S3). These relative release rates suggest a strong preferential kinetic reactivity of siderophores and synthetic chelators with Fe and Al in comparison to those of other elements.

This is related to high stability constants of the siderophore–metal complexes (siderophore_*p*=1_–metal_*q*=1_–proton_*r*=0_, β_110_ configuration) with the trivalent Fe(III) [log *k*_f_ = 25.1], Al(III) [log *k*_f_ = 16.4],^74^ divalent Fe(II) [log *k*_f_ = 11.4], compared for those of the alkaline-earth divalent metals Ca(II) [log *k*_f_ = 3.0] and Mg(II) [log *k*_f_ = 2.8], which are orders of magnitude lower.^75^ Siderophores form more stable surface complexes with Fe-oxide minerals than with non-Fe oxides, as measured by atomic force microscopy on the surface-bound ligands.^76^ Thus, in the initial steps of dissolution, siderophores are likely to be more strongly bound to Fe sites on the surface of minerals within basalt. Subsequently, the high stability/formation constants of the Fe(III)-siderophore surface complex favor destabilization of the Fe bound to the mineral lattice and accelerate the release of Fe.^29^ Upon preferential saturation of Fe surface sites by siderophores, Al(III) is then complexed. The preferential dissolution of these trivalent metals creates a Si-enriched surface layer, which dissolves to form H_4_SiO_4_ (aq) as the reaction product. This interpretation of chemical kinetic release of elements from the mineral surface as rate limiting for Fe-mineral dissolution by siderophores is further supported by the formation of etch pits on the surface of hornblende by siderophore attack^77^ and by experiments investigating basaltic glass dissolution in response to a single fixed concentration of desferrioxamine B mesylate.^36^ On the time scale and pH of our experiments, the base cations are released in excess of Fe (Figure 3A,D). This is expected due to the known rapid release of these cations during silicate dissolution, e.g., as rapid ion exchange reactions at the mineral surface.^78^ Mg dissolution from basalt is more responsive to chelator activity than that of Ca, consistent with the accelerated dissolution of ferromagnesian minerals—e.g., olivine [(Mg,Fe)_2_SiO_4_] in this basalt feedstock.^30^ The extent of base cation depletion depends on the rate of network-forming metal ion dissolution. Therefore, faster dissolution of Fe by siderophore attack combined with the high stability of the resulting metal-chelate complexes in solution: (a) prevents a back reaction,^23^ (b) limits the development of a protective oxidation ferric layer, and (c) creates a thicker surface reaction layer. Combined, these effects are expected to be accompanied by greater release of base cations^79^ (Figure 3B,E) and CDR, as demonstrated here (Figure 3C,F,G) and outlined in the equations for basalt-derived olivine above (Eq. 1a,b).
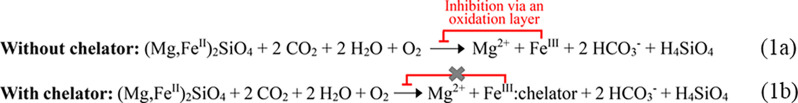


As indicated by the equations above, the molar ratio between released divalent cations and dissolved atmospheric CO_2_ in the form of bicarbonate is expected to be 1:2, whereas that of their equivalent charges should be 1:1. However, the weathering reactions took place in 1000 μM KCl solution, and basalt reactive surfaces and secondary clays (see Table S1 for the mineralogy) can exhibit negative charges. Consequently, solution K^+^ can be adsorbed onto basalt particles. Comparisons between end-of-experiment and initial K^+^ concentrations reveal that K^+^ adsorption onto basalt amounts to ∼12% of all K added to the initial solution (Figure S7). The resulting unbalanced negative charge (mainly Cl^–^ and some accessory negatively charged chelator anions; Figure S7) can counterbalance a fraction of Ca^2+^ and Mg^2+^ released in solution during basalt dissolution, as indicated by close to a 1:1 relationship between the sum of measured charge equivalents of Ca^2+^ and Mg^2+^ and the sum of measured HCO_3_^–^ and Cl^–^ (Figure S7). Accounting for the Ca^2+^ and Mg^2+^ counterbalanced by Cl^–^ resulted in divalent cation charge equivalent values in solution closely matching the 1:1 mixing line with measured HCO_3_^–^ (Figure 3C,F). These findings are consistent with geochemical theory and show that siderophore- and synthetic chelator-promoted dissolution of basalt powder in neutral solutions at equilibrium with atmospheric *p*CO_2_ results in a pattern consistent with weathering by carbonic acid.

### Soil Chelator Biotechnology Pathway for Accelerating EW?

We next undertook a series of soil incubation studies with the synthetic Fe-chelating agent K-EDDHA with the goal of understanding the feasibility of EDDHA application to (1) create conditions of Fe-starvation that force soil-native microbes to produce high-affinity siderophores to effectively compete with EDDHA for Fe and (2) directly accelerate rates of basalt mineral dissolution in soil.

**Figure 4:**
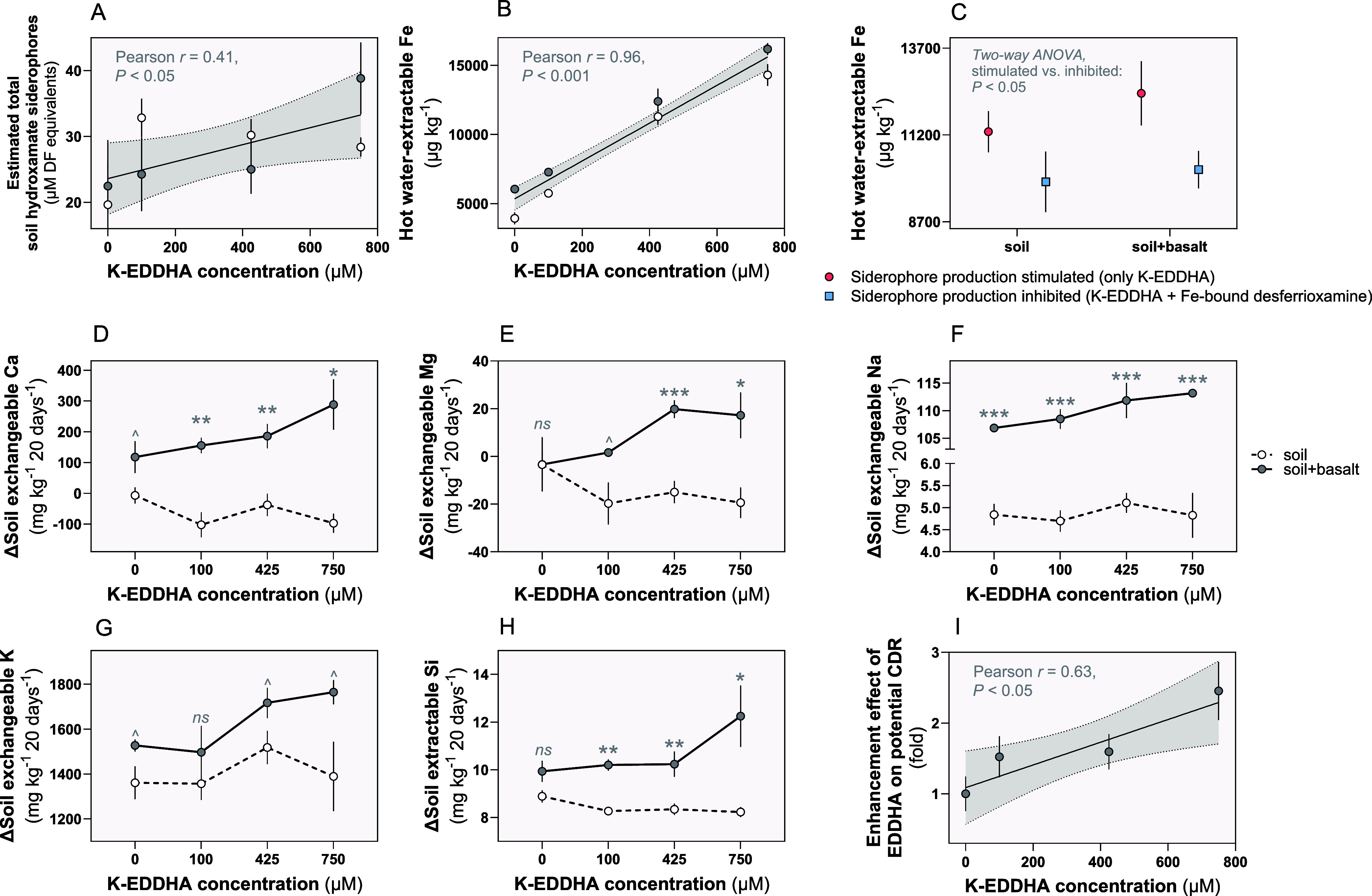
Effects of the chelating agent EDDHA on basalt weathering and carbon dioxide removal potential in soil. (A) Bioassay-based estimates of total hydroxamate concentrations in soil and soil + basalt incubated substrates after 10 days in response to EDDHA (Pearson correlation test, *P* < 0.05, *r* = 0.41). (B) Hot water-extractable Fe concentrations respond to EDDHA supporting the proposed role of EDDHA as a Fe-sequestering agent (measured 20 days after the start of incubation; Pearson correlation test, *P* < 0.001, *r* = 0.91). (C) Hot water-extractable Fe measured 20 days after the start of incubation is significantly lower in substrate incubations by prebound Fe-desferrioxamine siderophores. These results indicate a significant role of siderophores produced by the native community in dissolving Fe from soil and basalt. Difference in the exchangeable pools (1 M ammonium acetate extracts, pH 7.0) of (D) calcium (Ca), (E) magnesium (Mg), (F) sodium (Na), (G) potassium (K), and (H) silicon (Si) in substrate extracts after 20 days as a function of different levels of applied EDDHA. The differences between soil + basalt and soil for each EDDHA treatment show the effect of EDDHA on basalt weathering; two-tailed *t* tests were performed for each concentration (***P < 0.001, ** < 0.01, * < 0.05, ∧ < 0.10, ns > 0.10). (I) Potential enhanced weathering carbon dioxide removal (CDR) gain in response to EDDHA shows a significant linear relationship (Pearson correlation test, *P* < 0.05, *r* = 0.63) with the concentration of free EDDHA applied. CDR calculations are based on the following equation: Δcation_exch. soil+basalt_ – Δcation_exch. soil_ for the major divalent [Ca, Mg] and monovalent [Na, K] ions. Replication for each concentration and each soil treatment (soil vs soil + basalt) in replicates of three, *n* = 3. The non-SI units “mg kg^–1^ 20 days^–1^” are here preferred over the SI unit expression “mg kg^–1^ day^–1^” as to make clear that the delta values for soil nutrients are derived based on the difference in their values from day 20 to day 0 as per our incubation protocol. In this way, we also do not assume uniformity in the rate of change throughout the 20-day period.

Results of our incubation of field soils with variable concentrations of K-EDDHA (0, 100, 425, and 750 μM) revealed that the concentration of total hydroxamate siderophores in soil (including those in solution and those adsorbed^26^ onto organic and inorganic surfaces) produced by the microbial community increased linearly (Pearson correlation test, *P* < 0.05) in response to increasing EDDHA concentrations (Figure 4A). At the same time, EDDHA interacted with soil and basalt-derived Fe minerals, scavenging Fe and generating water-soluble Fe-EDDHA chelates, as seen by the strong linear trend between applied EDDHA and dissolved Fe (Pearson correlation test, *P* < 0.001) (Figure 4B). To assess if the observed EDDHA-mediated increase in microbial hydroxamate siderophores results in further chelation, we compared Fe availability in soils that had received either 425 μM EDDHA alone (stimulating siderophore production) or 425 μM EDDHA plus prebound Fe(III)-hydroxamates (inhibiting siderophore production). Our results showed significantly greater levels of soluble Fe (two-way ANOVA, *P* < 0.05) in soils with EDDHA-stimulated siderophore production in the absence of soluble Fe(III) compared to soils with siderophore production inhibited (Figure 4C) by the presence of bioavailable Fe(III)-hydroxamate complexes. This indicates that soil-applied EDDHA releases and complexes dissolved Fe from basalt and soil minerals and promotes the biosynthesis of siderophores by the belowground microbiome to further enhance Fe chelation.

To estimate the extent to which increased basalt dissolution with EDDHA increased CDR rates, we determined the relative increase in the bulk soil pool of ion-exchangeable Ca^2+^, Mg^2+^, Na^+^, and K^+^, (as products of weathering) following the incubation (20 days) of bulk soils in response to varied rates of EDDHA application (Figure 4D–H). Without EDDHA, there was a low rate of Ca release by basalt weathering (two-tailed *t* test, *P* < 0.10), whereas with EDDHA Ca release increased significantly relative to basalt-free control soil incubations (two-tailed *t* test, *P* < 0.01 and <0.05; Figure 4D). The increase in exchangeable Ca over the 20-day incubation period exhibited a significant linear trend with increasing EDDHA concentrations in soil + basalt samples (Pearson correlation test, *r* = 0.54, *P* < 0.05) but not in untreated soil (Pearson correlation test, *r* = −0.20, *P* ≫ 0.10), consistent with EDDHA stimulating EW and cation release. Similar trends were observed for exchangeable Mg, K, Na (Figure 4E–G), and Ti (Figure S5). The linear relationship between added chelator and accumulation of base cations at exchange sites in soil + basalt relative to untreated soil samples confirms alkalinity production that relates to enhanced CDR rate potential by EDDHA (Figure 3G). Soil incubations are expected to have *in situp*CO_2_ above atmospheric levels, resulting in the suppression of pH and a corresponding increase in silicate mineral dissolution rates. The resulting alkalinity production estimated from increases in base concentration at exchange sites in the soil incubations was ∼2.5-fold (Figure 4I) greater with EDDHA, whereas in our *in vitro* experiments at atmospheric *p*CO_2_ the increase was ∼2-fold (Figure 3G).

Current farming practices^80^ utilize the compound Fe(III)-EDDHA by adding it in a precomplexed form dissolved in water, to Fe-deficient arable lands at rates of 5–10 kg ha^–1^ year^–1^.^80^ Use of Fe(III)-EDDHA is preferred to that of other synthetic Fe-chelates, e.g., ethylenediamine tetraacetic acid (EDTA) and diethylenetriamine pentaacetic acid (DTPA), because it forms complexes that are unavailable for microbial uptake,^41^ is not readily metabolized by soil microorganisms,^41^ and its complexes with metals are more stable at the alkaline environments of the calcareous soils typically associated with Fe deficiency.^81,82^

**Figure 5:**
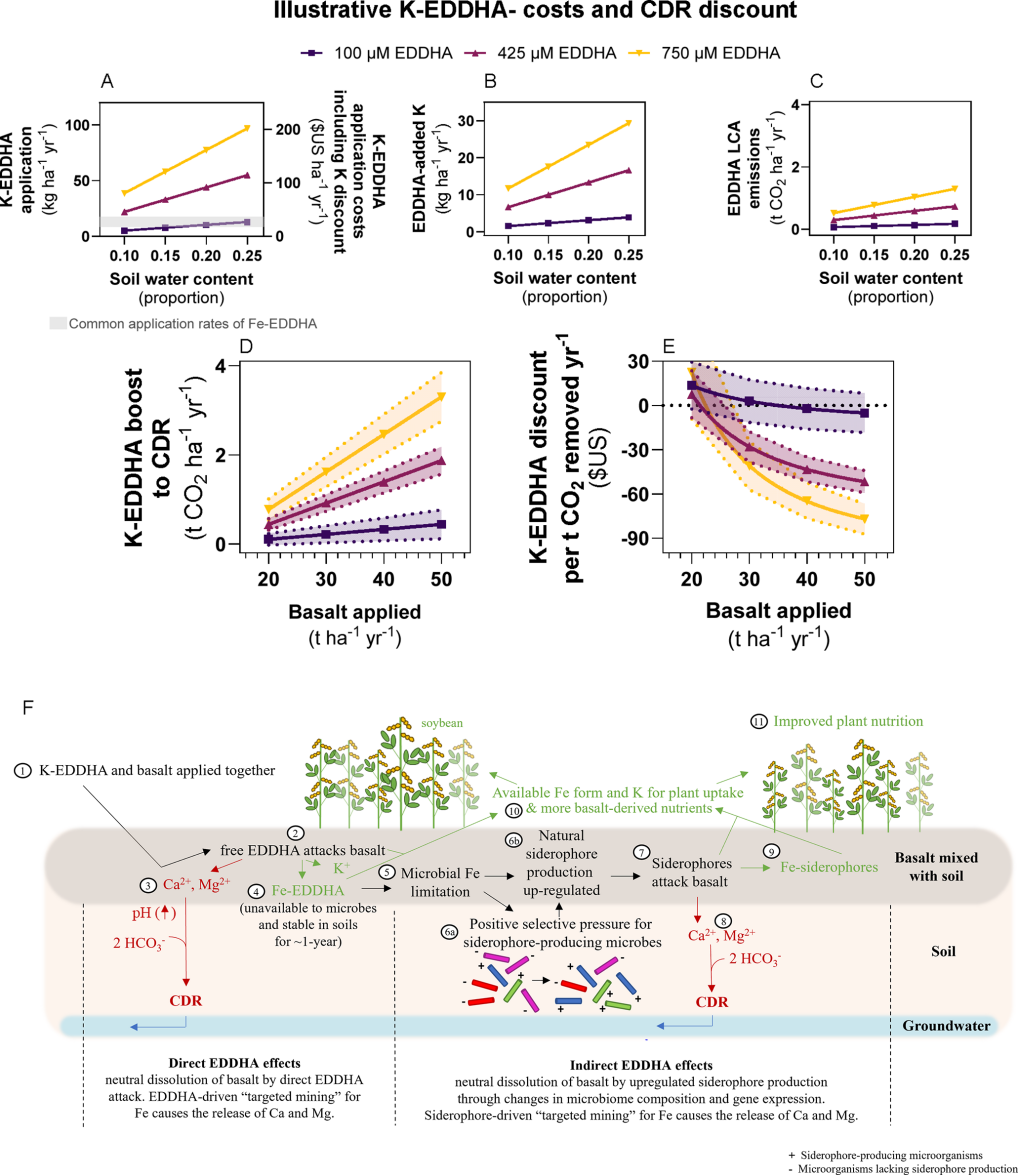
Cost-benefit analysis of EDDHA-driven enhanced weathering. (A) Rates of EDDHA application and costs for achieving target molar soil concentrations at different soil moisture levels. (B) Amount of K added by soil amendment with K-EDDHA. (C) Life-cycle assessment (LCA) emission of EDDHA. (D) CDR increase as a function of EDDHA and varied basalt application rates [experimental uncertainty and variability due to soil moisture (e.g., 10–25 wt %) are propagated; error bars show SEM]. (E) Cost per t CO_2_ year^–1^ captured by EW with or without chelating EDDHA; error bars show 1 standard deviation (SD) and are associated with the uncertainty of different soil moistures (propagation as specified in (D)). (F) Conceptual diagram illustrating how the application of Fe-free K-EDDHA increases EW and CDR through direct (EDDHA attack on basalt) and indirect effects (Fe-EDDHA effects on the microbiome and siderophore biosynthesis gene expression). The proposed treatment can increase crop yields and crop nutritional value for human consumption by improving the availability of Fe, adding additional K and increasing the supply of basalt-derived nutrients (e.g., Ca, Mg, Si, P).

In order to maximize the alkalinity addition from EDDHA-enhanced weathering, we propose the use of its KOH-neutralized [K_3_:EDDHA]^−^K^+^ potassium salt which provides the necessary chelating activity while avoiding the acidity of the addition of [H_4_:EDDHA]. To assess the potential economic improvement of EW-CDR by K-EDDHA, we undertook an initial cost–benefit assessment for its deployment using (1) average prices for bulk orders of K-EDDHA (assuming equal price to that of Fe-EDDHA), (2) estimated cost of capturing 1 t CO_2_ ha^–1^ year^–1^ with EW in the USA,^8^ (3) cradle-to-gate life-cycle assessment (LCA) CO_2_ emissions for EDDHA salt production,^83^ (4) field CDR rates measured from an EW field trial, located at the Energy Farm in the U.S. Corn Belt,^1,2^ and (5) benefits resulting from the additional supply of K and avoided fertilizer costs. These estimates are based on field-measured rates of EW-CDR multiplied by the relative effect (fold difference) of K-EDDHA on weathering as measured in our incubations with soils from the same field trials (Figure 4I). The resulting initial cost-benefit analyses (Figure 5) are thus illustrative of the potential for K-EDDHA as a biotechnology pathway for reducing CDR costs with EW. Field-level trials combining this chelator-based technology with EW are required to understand its real-world utility for a diverse set of agroecosystems.

Soil moisture is a key determinant of EDDHA costs in the field because the volume of soil water affects the amount of EDDHA necessary to reach particular target concentrations (Figure 5A). A target concentration of 750 μM EDDHA at soil moisture of 20% (typical for the early autumn when basalt is spread and plowed into soil after harvest) requires 77.5 kg of K-EDDHA ha^–1^ that would entail an associated cost of $209 ha^–1^. However, the input of K would offset the cost of required K fertilizer by around $38 ha^–1^ lowering the application price to $171 ha^−1^ as an additional 23.5 kg K ha^−1^ are added to fields (Figure 5B). The LCA emissions linked to EDDHA application at this rate would be ∼1.0 t of CO_2_ ha^–1^ (Figure 5C). The resulting boost in CDR (Figure 5D) at typical rates of basalt application (50 t ha^–1^ year^–1^) greatly offsets the stated LCA emissions.

Our provisional cost analysis suggests that field-applied K-EDDHA may decrease the cost of EW-CDR by up to $77 ± 10 per t CO_2_ ha^–1^ removed (Figure 5E) to make this technology increasingly competitive relative to other CDR strategies.^84^ By enhancement of the EW efficiency, EDDHA could reduce the basalt application rate required to achieve a target CDR rate. For example, using 750 μM EDDHA (and taking into account its associated LCA emissions), the ∼3.8 t CO_2_ ha^−1^ removed per year as measured in EW field trials in the U.S. Corn Belt could be achieved with an application rate of 29 t rock dust ha^-1^ year^−1^ rather than the 50 t basalt ha^−1^ year^−1^ needed without EDDHA. Similarly, assuming an EW application rate of 50 t basalt ha^–1^ year^–1^, an EDDHA amendment of 750 μM may increase field-measured CDR (3.8 t CO_2_ ha^–1^ year^–1^) by a further 3.3 ± 0.6 t CO_2_ ha^–1^ year^–1^ (Figure 5D). In fields subjected to EW, crop plants can acquire Fe from resulting Fe(III)–EDDHA complexes obtained from basalt, and the soil solution via root surface-localized iron reductase that splits the complex and reduces Fe(III) to Fe(II); the latter is then transported inside root cells.^42,85^ Thus, recycled EDDHA may further attack soil and/or basalt minerals containing Fe with the cycle repeating.^82^

Cobenefits for farmers and land managers of the combined effects of EDDHA include increasing availability of Fe to crops and an enhanced release of important inorganic nutrients, including P, K, Si derived from basalt weathering important for maintaining agricultural yields^4^ (Figure 5F). Importantly, Al and trace metals associated with toxicity to plants (Cd, Cr, As, Ni, and Pb) were not affected at the levels of EDDHA used in our experimental soil incubations (Table S2). Previous studies in Australian tropical soils have identified that the application of EDDHA may increase the levels of available Al^3+^ ions that are toxic to plants.^86^ However, EDDHA has a relatively lower affinity for Al^3+^ complexation compared to microbial siderophores (Figure 3A,D), and thus the formation of Al:EDDHA complexes will be highly dependent on the levels of Al-source minerals (e.g., clays). For instance, the tropical soils in this Australian study exhibited a range of clay with a total Al of 29–64%^86^ compared to our studied temperate agricultural soils with a mean total Al content of 4.9%. The only trace element that exhibited a consistent linear response to EDDHA treatment was copper (Cu; Table S2), which was increased by 20% with the highest EDDHA treatment. At the concentrations observed, Cu represents an important micronutrient to crop performance, necessary for several metabolic reactions including photosynthesis and respiration.^87^ Overall, it appears that achieving an EDDHA concentration (750 μM) in the soil solution to drive EW and promote CDR falls outside the range associated with Fe:EDDHA phytotoxicity (2000–4000 μM).^88^

Furthermore, in terms of its toxicity profile in animals, the Fe:EDDHA chelate has no bioaccumulation potential with low toxicity; oral doses of 2000 mg kg^–1^ body weight being required for causing 50% lethality among test animals (LD_50_).^89^ Similarly, the free chelate was not toxic to rodents at oral doses of up to 6000 mg kg^–1^ body weight.^90^ Consequently, the European Union Parliament authorized the use of EDDHA as an agricultural chelate in 2019.^91,92^

The practical application of EDDHA with EW and farmland requires a supply of commercial Fe-free potassium-EDDHA. This can be obtained by treating commercially available Fe:EDDHA products with a range of standard chemical approaches^41,93,94^ but those may be impractical for larger operations. Instead, the synthesis of Fe-free K-EDDHA would be more straightforward by omitting the addition of ferric salts and adding potassium hydroxide instead during the synthesis process.^95^ This would likely result in lowering the price of free EDDHA and its associated LCA CO_2_ emissions, as this process would omit the necessity for costly metal iron salt addition.

The proposed chelate-based technology would not change the total C sequestration potential resulting from basalt weathering. However, by increasing the reactive leached layer depth and avoiding the formation of a passivating oxidation layer/rind, chelators would increase the rate of C sequestration, important for reaching climate change mitigation goals within the required target time scales. Logistically, K-EDDHA could be added in a predissolved liquid form and sprayed across the field shortly after basalt application postharvest and before the latter is tilled in. This limits EDDHA–soil mineral interactions, thus avoiding destabilization of organo-mineral complexes and instead promoting direct EDDHA–basalt weathering interactions. Current practices advise that Fe-EDDHA is most beneficial to crops before the development of iron deficiency symptoms such as chlorosis^11^ during early to mid-spring.^10^ However, springtime application will likely require greater amounts of EDDHA as soil moisture in the U.S. Corn Belt is higher in spring than in early autumn.^96^ Given that the alleviation of Fe limitation on yields remains after EDDHA applications for up to 2 years,^97^ we propose that a single high-dose application of free EDDHA immediately after rock spreading will deliver the greatest benefits of EW and CDR on farmland.

## Supporting Information Available

Field experimental setup for rock grain bags at the Energy Farm, U.S. (Figure S1); genus-specific expression of the desferrioxamine and arthrobactin siderophore biosynthesis gene *desB* (Figure S2); elemental release from basalt in the absence or presence of high-affinity (siderophores) and low-affinity (citrate) iron chelators at different concentrations (Figure S3); dissolution of phosphorus from basalt in response to in vitro chelator-driven weathering (Figure S4); mobilization of titanium (Ti) at he exchange sites in response to EDDHA-driven weathering (Figure S5); sequential extraction procedure for incubated soil and soil + basalt samples in this study (Figure S6); charge balance in the chelator-free control, K-DF siderophore, and synthetic chelator K-EDDHA weathering solutions considering K adsorption onto basalt mineral surfaces from initial solutions (Figure S7); Hillhouse basalt mineralogy as outlined in Lewis et al.^30^ (Table S1); correlation table: change in soil exchangeable concentration of different elements between end (20 days) and start (0 days) of experimental incubation in soil and soil + basalt substrates in response to EDDHA concentrations (Table S2); modified *Arthrobacter* sp. JG-9 bioassay to measure hydroxamates in soil (Note S1) (PDF)
