# Supplementary material for: Iron Chelation in Soil: Scalable Biotechnology for Accelerating Carbon Dioxide Removal by Enhanced Rock Weathering
Source: Environ Sci Technol. 2024 Jun 24;58(27):11970–87. doi: 10.1021/acs.est.3c10146 (PMC11238546; doi:10.1021/acs.est.3c10146)
Supplement: Supplementary file 1 — es3c10146_si_001.pdf [file es3c10146_si_001.pdf]

Supporting Information

for

Iron chelation in soil: a scalable biotechnology for accelerating carbon dioxide removal by enhanced rock weathering

Dimitar Z. Epihov*, Steven A. Banwart, Steve P. McGrath, David P. Martin, Isabella L. Steeley, Vicky Cobbold, Ilsa B. Kantola, Michael D. Masters, Evan H. DeLucia & David J. Beerling

*Corresponding author: d.z.epihov@sheffield.ac.uk

Overall contents: 7 x Supporting Information Figures labelled Figure S1-S7, 2 x Supporting Information Tables labelled Table S1-S2, and 1 x Supporting Information Note. Details are as listed below:

Figure S1. Field experimental set-up for rock grain bags at the Energy Farm, US.
Figure S2. Genus-specific expression of the desferrioxamine and arthrobactin siderophore biosynthesis gene *desB*.
Figure S3. Elemental release from basalt in the absence or presence of high-affinity (siderophores) and low-affinity (citrate) iron chelators at different concentrations.
Figure S4. Dissolution of phosphorus from basalt in response to *in vitro* chelator-driven weathering.
Figure S5. Mobilization of titanium (Ti) at exchange sites in response to EDDHA-driven weathering.
Figure S6. Sequential extraction procedure for incubated soil and soil+basalt samples in this study.
Figure S7. Charge balance in the chelator-free control, K-DF siderophore, and synthetic chelator K-EDDHA weathering solutions considering K adsorption onto basalt mineral surfaces from initial solutions.
Table S1. Hillhouse basalt mineralogy as outlined in Lewis et al, 2021.
Table S2. Correlation table: change in soil exchangeable concentration of different elements between end (20 days) and start (0 days) of experimental incubation in soil and soil+basalt substrates in response to EDDHA concentrations.
Supplementary Note 1. Modified *Arthrobacter* sp. JG-9 bioassay to measure hydroxamates in soil.

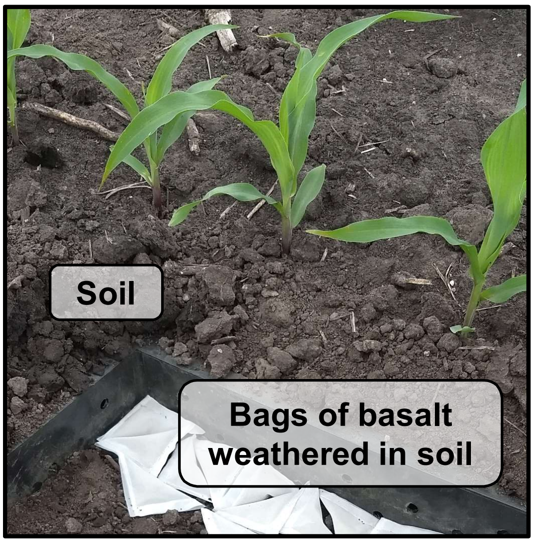

Figure S1. Field experimental set-up for rock grain bags at the Energy Farm, US. Heat-sealed polyethylene mesh bags (30 µm pore diameter) containing 4 grams of 53-90 µm fresh basalt grains were placed in the soil between plant rows. This bag deposition happened in control block 5 previously untreated with basalt. Bags were placed in a plastic washing basket pre-filled with soil with holes drilled on the side and holes on the bottom to allow for water flow and root interaction. This apparatus was covered with soil (not shown) and bags were left to weather for 1.5-years before they and surrounding soil were collected for omics analyses.

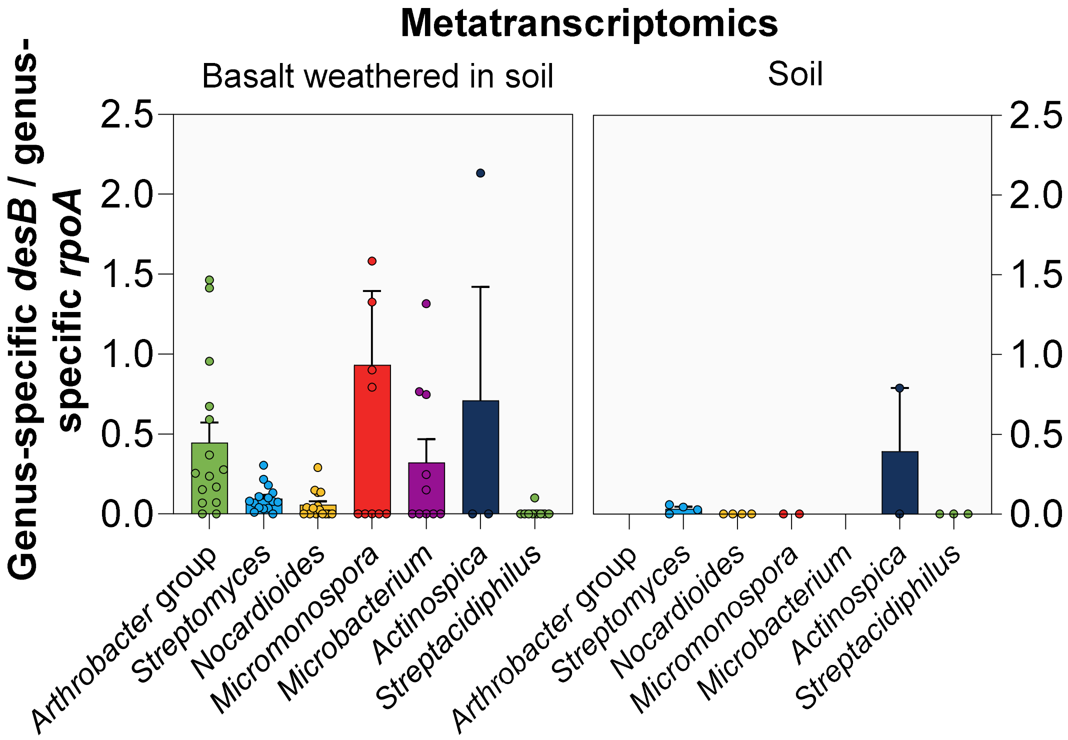

Figure S2. Genus-specific expression of the desferrioxamine and arthrobactin siderophore biosynthesis gene *desB* across abundant Actinobacteriota genera in field-weathered basalt and soil microbiomes. The metatranscriptomic expression data indicate that genera in weathered basalt microbiomes express *desB* to greater levels relative to their core metabolic activity (here exemplified by the housekeeping gene *rpoA*) than the same genera in soil. These data support the view that in addition to positive selective pressure recruiting siderophore-producing bacteria in basalt microbiomes relative to soil (Figure 1E), siderophore-producing bacteria are also actively utilizing the ability to produce siderophores during their life on basalt grains more so than members of the same genus in soil microbiomes. Error bars show SEM.

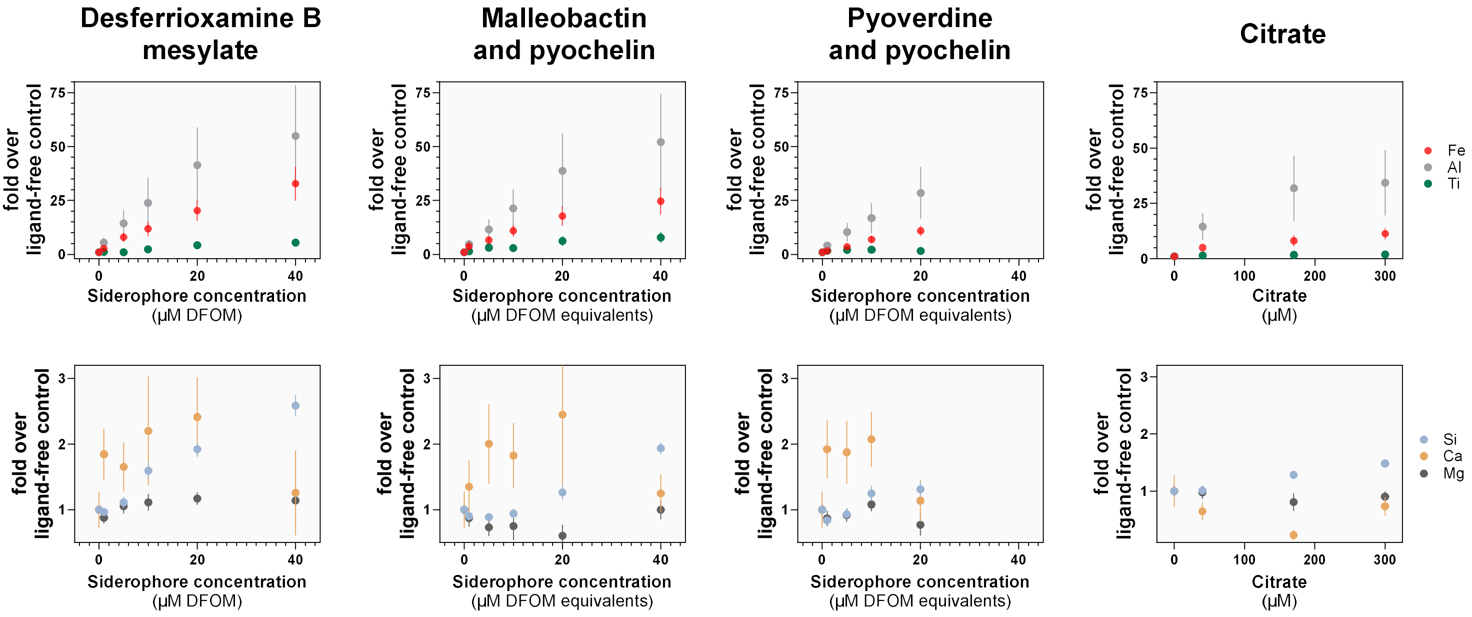

Figure S3. Elemental release from basalt in the absence or presence of high-affinity (siderophores) and low-affinity (citrate) iron chelators at different concentrations. Note that major divalent cations mainly Ca^{2+} and to a lesser extent Mg^{2+}, important for CDR, show patterns of increase only in response to siderophores but not citrate. The release of Fe, Al, and Ti from basalt is generally promoted by the presence of all tested chelators. There are no significant differences in the release of Mn from any of the chelators relative to control. Error bars show SEM.

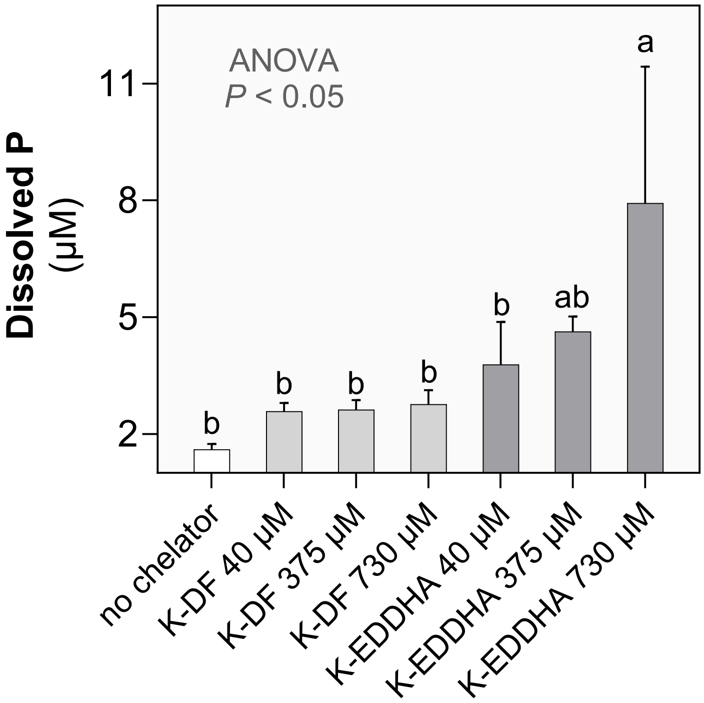

Figure S4. Dissolution of phosphorus from basalt in response to *in vitro* chelator-driven weathering. The K-EDDHA chelator releases greater amounts of phosphorus from basalt compared to dissolution without chelators and to that mediated by the siderophore K-desferrioxamine B mesylate. Multiple comparisons are based on ANOVA post-hoc Benjamini and Hochberg FDR multiple comparisons correction test.

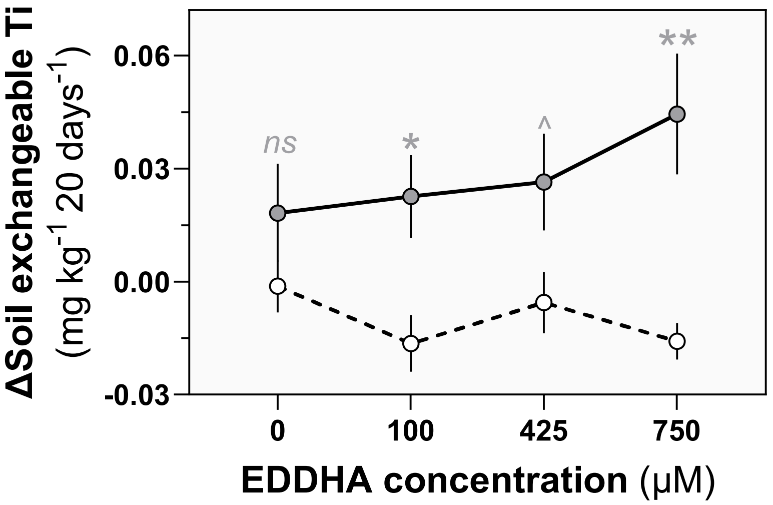

Figure S5. Mobilization of titanium (Ti) at exchange sites in response to EDDHA-driven weathering. These results suggest that only minimal levels of Ti are released in response to EDDHA-driven basalt weathering. At the observed concentrations, this release should not strongly affect mass balance approaches for estimating weathering rates (e.g., TiCat) that utilize Ti as a stable tracer for the amount of basalt in soil+basalt mixtures typical to soil samples in EW trials. Statistical results are based on two-tailed t-tests (** $P < 0.01$, * < 0.05, ^ < 0.10, *ns* > 0.10) comparing soil (white-filled circles) with soil+basalt (grey-filled circles) means for each level of EDDHA applied (no EDDHA i.e., 0 and 100, 425, and 750 µM EDDHA). Error bars show SEM.

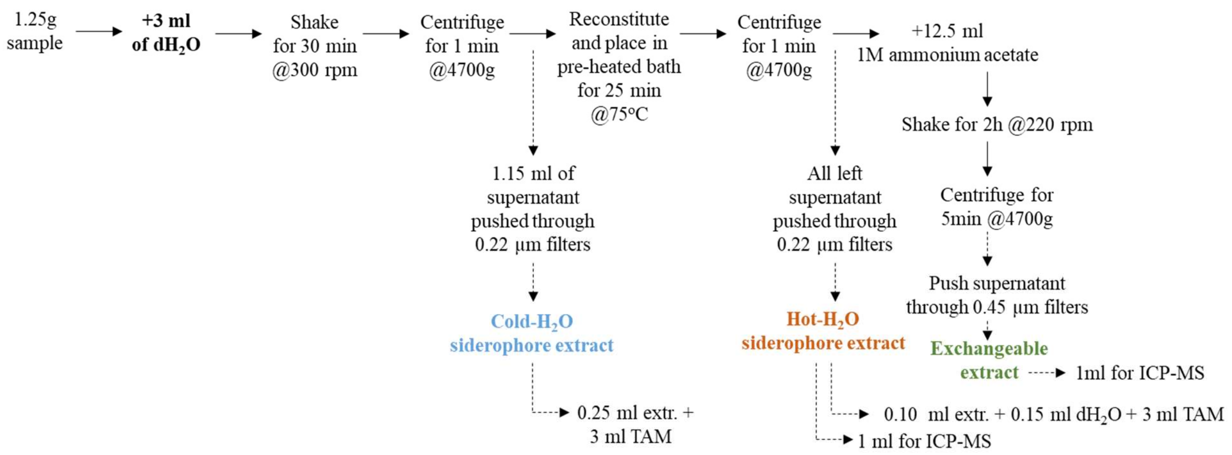

Figure S6. Sequential extraction procedure for incubated soil and soil+basalt samples in this study. All the soil samples were extracted with 3 ml of ultrapure water as shown in Figure S6. After shaking and centrifuging, an aliquot of the cold-water extraction in the supernatant (i.e. room-temperature) was filtered and used for further assays. The remaining contents of the tube were reconstituted with the left-over water, and the sample heated up in a water bath as specified in the figure above. Note that no further water was added. After incubation and centrifuging, all remaining hot-water extract in the supernatant was taken out, filtered and used for further assays. Following this, the soil pellet was resuspended and extracted with 12.5 ml 1M ammonium acetate buffered at pH 7.0. Hydroxamate siderophore concentration was determined using the hydroxamate auxotrophic bacterium *Arthrobacter* sp. JG-9 (now *Microbacterium flavescens* JG-9). The bacterium was grown in the terregens factor assay medium (TAM) medium[1] using soil extract of specified volume. Standard curve was based on *Arthrobacter* sp. JG-9 growth (measured by OD_{600}) in TAM medium with added desferrioxamine B mesylate at known concentrations. The hot water extracts and exchangeable extracts were diluted and acidified to 2% nitric acid and send for ICP-MS together with appropriate blanks.

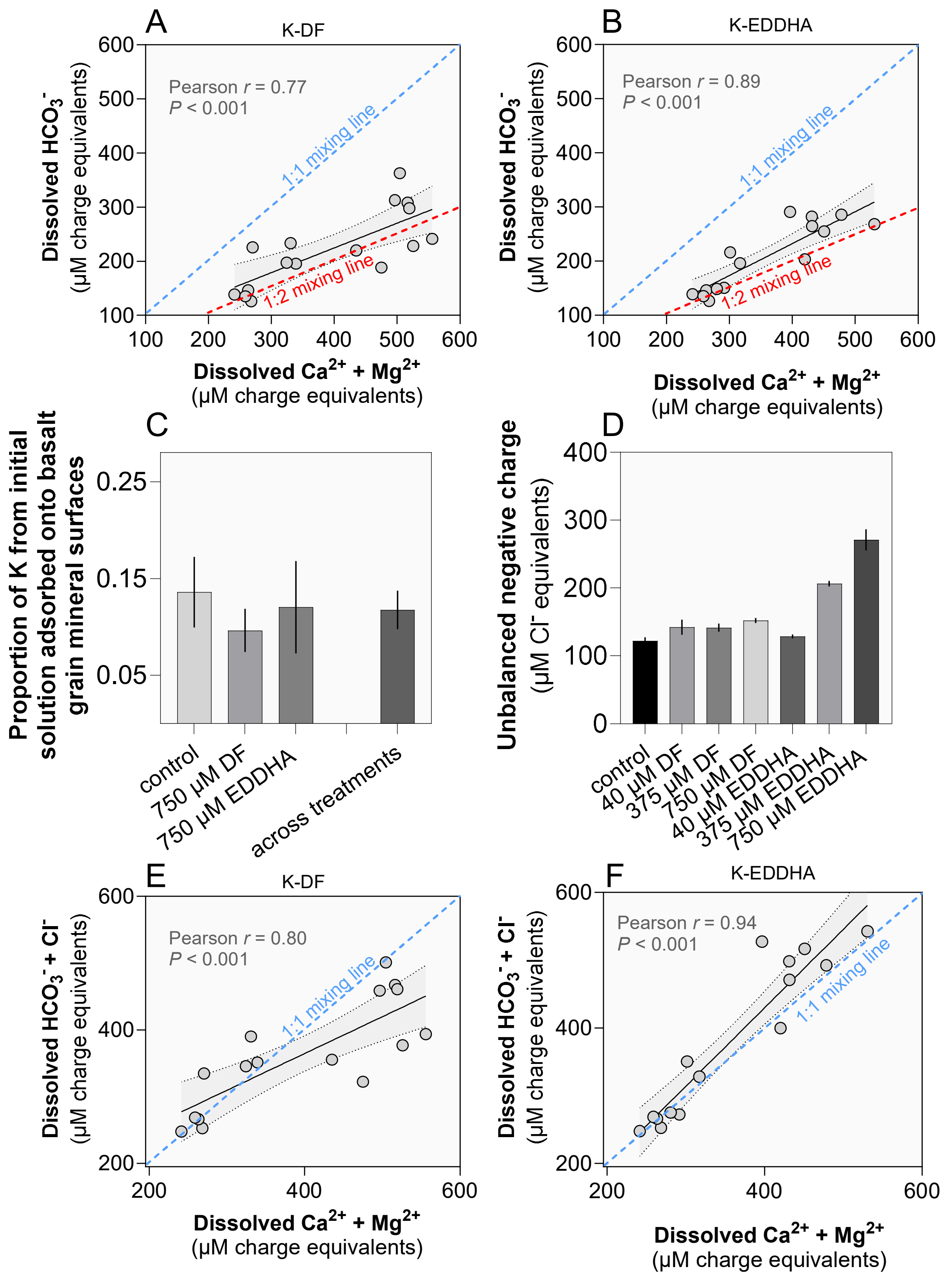

Figure S7. Charge balance in the chelate-free control, K-DF siderophore, and synthetic chelate K-EDDHA weathering solutions considering K adsorption onto basalt mineral surfaces from initial solutions. A,B – Charge equivalents Ca^{2+} + Mg^{2+} plotted against HCO_3^-. The plots show a relationship more closely matching the 1:2 mixing line than the 1:1 line. These data are suggestive of other negatively charged ions different than bicarbonate. **C,D.** Proportion of K^+ adsorbed onto basalt surfaces and unbalanced negative charge. Since weathering reactions took place in 0.001M KCl solution (equivalent to 1000 µM K^+ and 1000 µM Cl^-), adsorption of K^+ onto basalt mineral surfaces (negative charge of reactive surfaces or basalt-derived clays) can leave unbalanced Cl^- in the weathering solution. Moreover, added chelators (particularly K_3^+:$EDDHA^{3-}$) contain a large amount of K^+ which upon the

complexation of $EDDHA^{3-}$ with basalt-derived metals may also remain free and unbalanced in solution. Unbound free $EDDHA^{3-}$ in solution is negatively charged and similarly to Cl^- can act as a counterbalance anion for Ca^{2+} and Mg^{2+} released by basalt dissolution. Therefore, we assumed that the measured average proportion of K^+ adsorbed calculated as:

$$\text{Mean proportion } K_{adsorbed} = (\text{mean } K_{initial\ solution} - \text{mean } K_{end\text{-}of\text{-}experiment})/\text{mean } K_{initial\ solution}.$$

The calculated mean proportion of K adsorbed is then used to estimate the sample-specific K^+ adsorbed onto basalt which would be equal to Cl^-/chelator$^-$ left in solution and free to balance cation release as per the formula below:

$$K_{adsorbed} = \text{unbalanced neg. charge } (Cl^- \text{ eq.}) = [K_{end\text{-}of\text{-}experiment} /(1 - \text{mean prop. } K_{adsorbed})] - K_{end\text{-}of\text{-}experiment.}$$

E,F. Charge equivalents $Ca^{2+} + Mg^{2+}$ plotted against $HCO_3^- + Cl^-$ eq. Note that upon the inclusion of the unbalanced negative charge (measured as Cl^- equivalents) the negative charge of the final weathering solution exhibits a close match to the 1:1 mixing line with the positive charge equivalents provided by the $Ca^{2+} + Mg^{2+}$.

The values for ***"$Ca^{2+} + Mg^{2+}$ (µM charge equivalents) after accounting for that balanced by Cl^-"*** as they appear in the main text Figure 3C,F are calculated as:

$$Ca^{2+} + Mg^{2+}{}_{after\ accounting\ for\ Cl-} = Ca^{2+} + Mg^{2+} \text{ (charge equivalents)}_{measured} - \text{unbalanced neg. charge } (Cl^- \text{ eq.}).$$

Table S1. Hillhouse basalt mineralogy as outlined in Lewis et al, 2021.[2]

Mineral	Formula	wt. %
Plagioclase	$(Ca_{0.56}Na_{0.44})Al_{1.56}Si_{2.44}O_8$	34.6
Augite	$(Na_{0.02}Ca_{0.73}Fe_{0.25})(Mg_{0.83}Ti_{0.04}Fe_{0.02})(Al_{0.10}Si_{0.90})_2O_6$	32.7
Olivine (64% forsterite, 36% fayalite)	$Mg_{1.28}Fe_{0.72}SiO_4$	14.7
Spinel	$(Mg_{0.23}Al_{0.57}Ti_{0.14})(Cr_{0.67}Fe_{1.44})O_4$	4.5
Analcime	$Na_{0.94}K_{0.02}Ca_{0.04}Al Si_2O_6$	7.1
Chlorite-Smectite (*clay*)	$(Mg_{0.52}Fe_{0.32}K_{0.03}Na_{0.27}Ca_{0.1})Al(Si_{3.58}Al_{0.42})O_{10}(OH)_8$	5.2
Ilmenite	$(Fe_{1.42}Ti_{0.55}Mn_{0.03})O_3$	0.6
Biotite	$K(Mg,Fe)_3(AlSi_3O_{10})(OH)_2$	0.4
Calcite	$CaCO_3$	0.2
Apatite	$Ca_5(PO_4)_3(F,Cl,OH)$	0.1

Table S2. Correlation table: change in soil exchangeable concentration of different elements between end (20 days) and start (0 days) of experimental incubation in soil and soil+basalt substrates in response to EDDHA concentrations.

ΔElement (20d-0d)	soil		soil+basalt	
	Pearson's *P*	Pearson's *r*	Pearson's *P*	Pearson's *r*
Fe	***	0.924	***	0.987
Ti	*ns*	-0.192	*ns*	0.448
Al§	*ns*	-0.327	*ns*	0.128
Ca	*ns*	-0.200	*	0.535
Mg	*ns*	-0.290	*	0.571
K	*ns*	0.170	*	0.611
Na	*ns*	0.104	*ns*	0.433
Si	*ns*	-0.345	^	0.491
Cd	*ns*	-0.164	*ns*	0.035
Cr	*ns*	-0.015	*ns*	0.096
Ni	**	0.685	*ns*	0.191
Cu	***	0.711	**	0.818
As	*ns*	-0.004	*ns*	0.398
Cs	*ns*	-0.071	*ns*	0.195
Tl	*ns*	-0.091	*ns*	0.153
Pb	**	-0.597	*ns*	-0.047
P	*ns*	-0.180	*ns*	0.191
S	*ns*	0.002	*ns*	0.133
Sr	*ns*	-0.090	*ns*	0.479
Rb	*ns*	-0.019	*ns*	0.399

§Based on soil hot-water extraction as exchangeable Al was below detection limits.
Pearson correlation test, *** $P < 0.001$, ** < 0.01, * < 0.05, ^ < 0.10, *ns* > 0.10.

Supporting Information Note 1. Modified *Arthrobacter* sp. JG-9 bioassay to measure hydroxamates in soil.

To determine the hydroxamate concentration of our soil and soil+basalt cold water and hot water extracts, approximately 12 µl of *Arthrobacter* sp. JG-9 (now *Microbacterium flavescens* JG-9, acquired from NCIMB under number NCIMB 9471) cell suspension derived from cells cultured in +DF TAM were harvested by centrifugation, supernatant removed and then washed with -DF TAM several times and diluted to obtain OD_{600} = 0.17. The cultures were prepared with soil extract : medium ratios as specified in **Figure S6** and were incubated in the dark at 27°C on an end-to-end shaker at 15 rpm for 48h. The Terregens factor assay medium (TAM) medium[1] was prepared by adding to 1L ultrapure dH_2O the following chemicals: 2.0 g casamino Acids, 1.0 g yeast extract, 1.0 g D-glucose, 1.0 g ammonium hydrogen citrate, 2.0 g K_2HPO_4, 0.2 g $MgSO_4.7H_2O$, 0.03 g $FeCl_3.6H_2O$. The medium was adjusted to pH 7.0 using 1M KOH (2.2442 g KOH in 40 ml ultrapure water). The resulting medium was autoclaved and the concentration of 0.2 µg desferrioxamine B mesylate (DF) ml^{-1} medium was achieved by supplementing the cooled medium with 1.522 ml of filter-sterilised 200 µM DF stock solution in ultrapure water.

To convert between hydroxamates present in soil solution and total hydroxamates in soil (including hydroxamates adsorbed onto clay particles and organic matter complexes), we spiked soil samples with pre-bound Fe:desferrioxamine B mesylate complex (Fe:DF) to achieve final concentration of 20 µM. The difference in *Arthrobacter* sp. JG-9 growth converted to Fe:DF equivalents (using a standard curve as described in **Figure S6**) in cultures containing extract from samples with or without added 20 µM Fe:desferrioxamine complex was assumed to indicate the available (non-adsorbed) concentration of the added spike. The adsorbed hydroxamate level was calculated as the difference between the added (20 µM Fe:desferrioxamine) and the measured (non-adsorbed) siderophore concentration. From these the % adsorption was calculated and applied to measurements of non-adsorbed hydroxamates based on *Arthrobacter* sp. JG-9 growth using extract from experimental samples in order to estimate the total pool of hydroxamates in soil. *Arthrobacter* sp. JG-9 did not grow in TAM medium containing Fe:EDDHA complex at 0.1 µg ml^{-1} confirming that the presence of EDDHA in soil extracts did not affect its growth directly but only through the effects of EDDHA on hydroxamate siderophore biosynthesis by the native microbiome.

Supplementary references

(1) Fekete, F. A. Assays for Microbial Siderophores. In *Iron Chelation in Plants and Soil Microorganisms*; Barton, L., Hemming, B., Eds.; Academic Press, Inc., 1993; pp 399–418.

(2) Lewis, A. L.; Sarkar, B.; Wade, P.; Kemp, S. J.; Hodson, M. E.; Taylor, L. L.; Yeong, K. L.; Davies, K.; Nelson, P. N.; Bird, M. I.; Kantola, I. B.; Masters, M. D.; DeLucia, E.; Leake, J. R.; Banwart, S. A.; Beerling, D. J. Effects of Mineralogy, Chemistry and Physical Properties of Basalts on Carbon Capture Potential and Plant-Nutrient Element Release via Enhanced Weathering. *Appl. Geochemistry* **2021**, *132* (May), 105023. https://doi.org/10.1016/j.apgeochem.2021.105023.
